# Control of arbuscule development by a transcriptional negative feedback loop in *Medicago*

**DOI:** 10.1038/s41467-023-41493-2

**Published:** 2023-09-16

**Authors:** Qiang Zhang, Shuangshuang Wang, Qiujin Xie, Yuanjun Xia, Lei Lu, Mingxing Wang, Gang Wang, Siyu Long, Yunfei Cai, Ling Xu, Ertao Wang, Yina Jiang

**Affiliations:** 1 School of Life Sciences, East China Normal University, 200241 Shanghai, China; 2 National Key Laboratory of Plant Molecular Genetics, CAS Center for Excellence in Molecular Plant Sciences, Institute of Plant Physiology and Ecology, Shanghai Institutes for Biological Sciences, Chinese Academy of Sciences, 200032 Shanghai, China

**Keywords:** Arbuscular mycorrhiza, Plant molecular biology

## Abstract

Most terrestrial plants establish a symbiosis with arbuscular mycorrhizal fungi (AMF), which provide them with lipids and sugars in exchange for phosphorus and nitrogen. Nutrient exchange must be dynamically controlled to maintain a mutually beneficial relationship between the two symbiotic partners. The WRI5a and its homologues play a conserved role in lipid supply to AMF. Here, we demonstrate that the AP2/ERF transcription factor MtERM1 binds directly to AW-box and AW-box-like *cis*-elements in the promoters of *MtSTR2* and *MtSTR*, which are required for host lipid efflux and arbuscule development. The EAR domain-containing transcription factor MtERF12 is also directly activated by MtERM1/MtWRI5a to negatively regulate arbuscule development, and the TOPLESS co-repressor is further recruited by MtERF12 through EAR motif to oppose MtERM1/MtWRI5a function, thereby suppressing arbuscule development. We therefore reveal an ERM1/WRI5a–ERF12–TOPLESS negative feedback loop that enables plants to flexibly control nutrient exchange and ensure a mutually beneficial symbiosis.

## Abstract (web summary)

Zhang et al. report that Medicago AR2/ERF transcription regulators WRI5a-ERM1-ERF12 form a transcriptional negative feedback loop to coordinate arbuscular lipid supply, enabling the maintenance of a stable, reciprocally beneficial symbiosis.

## Introduction

The arbuscular mycorrhizal (AM) association is ubiquitous in the plant kingdom; it helps host plants efficiently obtain inorganic soil nutrients such as phosphorus and enhances their resistance to biotic and abiotic stress^1–5^. AM fungi are lipid auxotrophs whose asexual life cycle depends on lipids derived from the host plant to preserve the mutualistic relationship^6–9^. Lipid transfer to AM fungi is present in early-diverging *Marchantia paleacea*, suggesting that it may have supported the earliest AM symbioses, which arose over 460 million years ago^10,11^.

Lipids are essential components of all living cells whose uptake and metabolism are controlled by a conserved pathway in most plants. The APETALA2/Ethylene-responsive factor (AP2/ERF)-domain transcription factor WRI1 (WRINKLED 1) is a well-characterised transcriptional activator of the fatty acid biosynthetic pathway that binds directly to the AW-box [CnTnG(n)_7_CG] in target gene promoters^12–15^. WRI1 homologues in the WRINKLED1-like subfamily of AP2 family transcription factors, such as MtWRI5a/b/c in *Medicago truncatula*, CBX1 in *Lotus japonicus*, OsWRI5a/b in *Oryza sativa*, and MpaWRI in the liverwort *M. paleacea*, are required for arbuscule development, and they function in lipid biosynthesis and transfer during the mycorrhizal symbiosis^11,16–18^.

Arbuscules are highly branched structures that form in the inner cortex cells of plant roots. They are surrounded by a continuous host-derived peri-arbuscular membrane that provides a broad interface for bidirectional nutrient exchange^10^. AM-specific transporters, such as the phosphate transporter PT4, the putative lipid transporter STR (stunted arbuscule, an ATP binding cassette transporter), and the potential sugar exporter MtSWEET1b, are required for arbuscule development and nutrient exchange^6,19–21^. WRI5a and CBX1 bind directly to AW-box and/or CTTC motifs in target gene promoters to simultaneously activate the host phosphate-uptake pathway and arbuscular lipid provision during the AM symbiosis. WRI5a and CBX1 therefore appear to function as a molecular switch that regulates bidirectional nutrient exchange during the AM symbiosis^16,17^. Several GRAS family transcription factors, such as DELLA, RAM1 (Required for Arbuscular Mycorrhization 1), NSP2 (Nodulation Signaling Pathway 2), and MIG1 (Mycorrhiza-Induced GRAS 1), are also involved in the mycorrhizal symbiosis^22–27^. A study of an interconnected network of hundreds of rice transcription factors revealed an AM symbiosis transcriptional regulatory network centred on plant phosphate starvation response (PHR) transcription factors, suggesting that a dense network of feed-forward loops integrates mycorrhizal symbiosis, nutrient capture, and plant development^18,28–30^.

Highly branched arbuscules are terminally differentiated structures with a relatively short life span of approximately 3–7 days from branching to maturation and degradation^31,32^. To maintain a stable and mutually beneficial relationship during the AM symbiosis, plant root cortical cells face continuous challenges at each stage of arbuscule development and nutrient exchange^33^. Here, we demonstrate that two ERF family transcription factors, ERM1 and ERF12, together with WRI5a, can form a negative feedback loop that functions as a central module for fine-tuning of arbuscule development in *Medicago*. We propose that this may serve to limit excess resource expenditure and stabilise the mycorrhizal symbiosis.

## Results

### MtSTR2 is required for arbuscule development and participates in lipid export

**Fig. 1:**
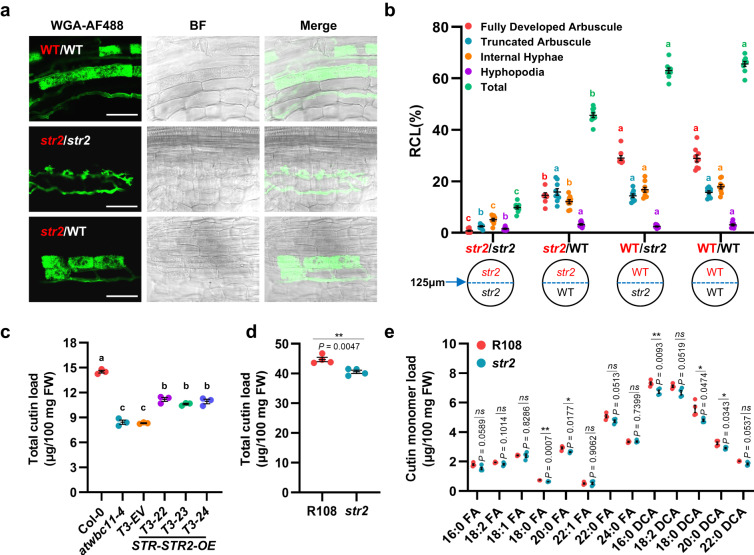
MtSTR2 is required for arbuscule development and contributes to cutin content. **a**–**b** Images of WGA-AF488-stained arbuscules (**a**) and quantification of *R. irregularis* colonisation level (**b**) of the tester plant roots at 6 weeks post-inoculation with *R. irregularis* (wpi) in a nurse system (*str2* and wild-type [WT] plants grown in the presence of a *str2* or WT nurse plant). BF: Bright-field. Scale bar, 50 μm. The tester plant is labelled in red, and the nurse plant is labelled in white and black in (**a**) and (**b**), respectively. **c** Total cutin load of *Arabidopsis* Col-0, *atwbc11-4* mutants, empty vector controls (T3-EV), and *STR-STR2* co-overexpressing (T3-22, T3-23, and T3-24) transformed *atwbc11-4* mutant T3 lines. **d**–**e** Total cutin load (**d**) and cutin monomer load (**e**) of roots from 4-week-old WT (R108) and *str2* seedlings grown in sand/perlite (1:1) without mycorrhizal fungal infection. FW, fresh weight. Statistics: Individual data points and mean ± SE are shown. Different letters (**b**, **c**) indicate significant differences (One-way ANOVA, Duncan’s multiple range test, *P* < 0.05). Exact *P* values are provided in Source Data. **b** *n* = 9 biologically independent plants; **c** *n* = 3 biologically independent samples. **d**–**e** *n* = 4 biological replicates from 20 plants. Two-sided Student’s *t* test. *P < 0.05; **P < 0.01; ns not significant.

The two half-size ABCG transporters STR and STR2 interact with each other in *Medicago*^20^, and STR is required for export of plant-derived lipids in arbuscules^6,8,9^. Here, a bimolecular fluorescence complementation (BiFC) assay showed that STR2 formed heterodimers with STR but did not homodimerise in *Nicotiana benthamiana* leaves, implying that STR2 and STR are functionally dependent on one another (Supplementary Fig. 1). To examine the function of *STR2* during the AM symbiosis, we identified the *STR2*-defective mutant *str2* (*NF11835*), which contains a *Tnt-1* insertion within the *STR2* genomic sequence (Supplementary Fig. 2a–c). The *str2* mutant showed significantly lower levels of AM colonisation and tiny, stunted arbuscules compared with wild-type R108 at 6 weeks post-inoculation (wpi) with *Rhizophagus irregularis*; its phenotypes were similar to those of STR2 RNA interference (RNAi) roots (Supplementary Fig. 2d, e)^20^. Mycorrhizal colonisation and *PT4* expression in *str2* were complemented by *STR2* driven by its native promoter (Supplementary Fig. 2c–e). Conversely, *str2* formed fully developed arbuscules with increased *PT4* expression when it was cultivated with a wild-type ‘nurse plant’ (A17) after inoculation with *R. irregularis* (Fig. 1a, b and Supplementary Fig. 2f–h). These results were similar to those observed with *RAM2* and *STR*, which are involved in lipid biosynthesis and transfer^6,16^. Thus, STR2 is required for arbuscule development and may participate in lipid export during the AM symbiosis.

In *Arabidopsis*, the half-size ABCG transporter AtABCG11/WBC11 (At1g17840) functions in the secretion of cutin and wax, mainly comprising lipids and their derivatives, from the leaf epidermis to the cuticle^34^. *atwbc11-4* plants displayed organ fusions, stunted growth, and reductions in wax and cutin monomers^34^. Although STR/STR2 orthologs were not identified in non-mycorrhizal *Arabidopsis*^20^, co-overexpression of *STR*-*STR2* in *Arabidopsis atwbc11-4* plants significantly increased cuticular cutin content, especially content of 16:0-fatty acid (FA), 18:2-FA, 18:3-FA, 18:2-dicarboxylic acid (DCA), and 18:1-DCA monomers (Fig. 1c and Supplementary Fig. 3a, b), and it partially complemented the reduced plant height phenotype caused by organ fusion (Supplementary Fig. 3c, d). Furthermore, concentrations of cutin monomers (especially 18:0 FA, 20:0 FA, 16:0-α,ω-DCA, 18:0-DCA, and 20:0-DCA monomers) were significantly lower in roots of *str2* than in those of the wild type, whereas total FA content did not differ significantly (Fig. 1d, e and Supplementary Fig. 4). These data suggest that both STR2 and STR contribute to export of specific lipid constituents.

Taken together, the above data suggest that STR2 is required for arbuscule development and participates in lipid export by forming a heterodimer with STR.

### ERM1 activates expression of *STR* and *STR2* via AW-box and AW-box-like motifs

**Fig. 2:**
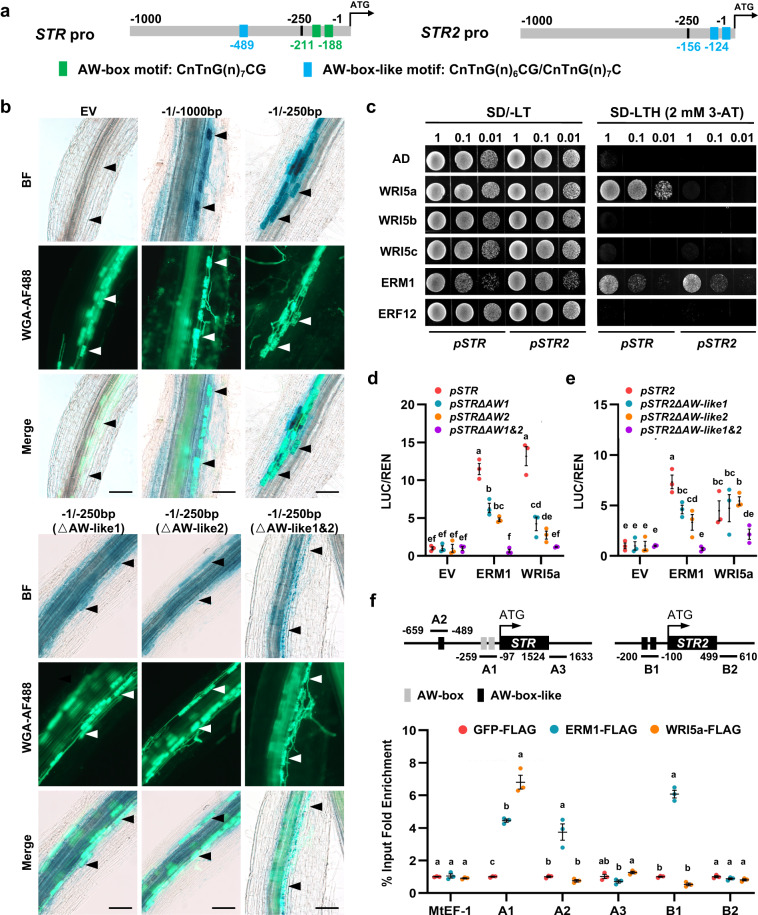
ERM1 activates the expression of *STR2* via two AW-box-like motifs. **a** Schematic of 1-kb *STR* and *STR2* promoter regions (upstream of the ATG start codon) with AW-box motifs (in green) and AW-box-like motifs (in blue). **b** Localisation of *GUS* expression in mycorrhized *M. truncatula* hairy roots (A17 background) driven by different lengths (1000 or 250 bp upstream of the ATG start codon) or forms (single or double deletion of the AW-box-like motifs) of the *STR2* promoter at 4 wpi. Bright-field (BF), fluorescence microscopy (WGA-AF488), and overlap (Merge) images are shown from top to bottom for GUS staining and fungal structures. Arrowheads indicate cells containing arbuscules. Scale bars, 100 μm. All samples underwent GUS staining for 0.5 h. Experiments were repeated 3 times with similar results. **c** ERM1 interacts with the *STR* and *STR2* promoters in a yeast one-hybrid (Y1H) assay. Y1H was performed using 250-bp *STR/STR2* promoters as baits and WRI5a, WRI5b, WRI5c, ERM1, and ERF12 as prey. The numbers on top of each photograph indicate the different concentrations of yeast cells. SD/-LT, synthetic dropout (SD) medium lacking leucine and tryptophan; SD/-LTH, SD medium lacking leucine, tryptophan, and histidine. 3-AT, 3-amino-1,2,4-triazole. **d–e** In vivo transcriptional activation assay using a dual-luciferase system showing the activation of the 250-bp *STR* (**d**) and *STR2* (**e**) promoters with various deletions of AW-box/AW-box-like elements by ERM1 or WRI5a, which acted as effectors. **f** ChIP-qPCR analysis of ERM1 and WRI5a binding to *STR* and *STR2* promoters. Transgenic roots expressing ERM1-FLAG, WRI5a-FLAG, and GFP-FLAG (negative control) driven by the *Ubiquitin* promoter were used in anti-FLAG ChIP experiments at 6 wpi with *R. irregularis*. qPCR was performed using primers surrounding the AW-box (-like) motifs (A1, A2, and B1) in the *STR* and *STR2* promoters or excluding the AW-box (-like) motifs (A3, B2) in the coding regions. *MtEF-1* is a negative control. Statistics: Individual data points and mean ± SE are shown. Different letters (**d–f**) indicate significant differences (One-way ANOVA, Duncan’s multiple range test, *P* < 0.05). Exact *P* values are provided in Source Data. **d–f** *n* = 3 biologically independent samples.

The *Medicago* AP2/ERF-domain transcription factor WRI5a acts as a positive regulator of lipid biosynthesis and transfer by binding to the AW-box [CnTnG(n)_7_CG] in the promoters of *STR*, *PT4*, and fatty acid biosynthesis genes during the mycorrhizal symbiosis^16^. Intriguingly, two AW-box-like motifs [CnTnG(n)_6_CG/(CnTnG(n)_7_C]^35^ (−124/−156 bp upstream of the ATG start codon) are present in the 2-kb *STR2* promoter region (Fig. 2a). We found that *STR2* expression was induced in arbuscule-containing cells and that a 250-bp *STR2* promoter fragment with two AW-box-like motifs was sufficient to drive GUS expression specifically in arbuscule-containing cells (Fig. 2b and Supplementary Fig. 5). Single or double deletion of the two AW-box-like motifs in the 250-bp *STR2* promoter reduced the intensity of GUS signals in arbuscule-containing cells (Fig. 2b and Supplementary Fig. 5). These data suggested that both AW-box-like motifs are necessary to specifically induce *STR2* in arbuscule-containing cells.

WRI5a bound to the *STR* promoter but not to the *STR2* promoter in a yeast one-hybrid (Y1H) assay^16^ (Fig. 2c). We therefore proposed that additional AP2/ERF transcription factors might participate in transcriptional activation of *STR2*. After searching the *Medicago truncatula* Gene Expression Atlas (https://medicago.toulouse.inrae.fr/MtExpress), we identified two more AP2/ERF-domain transcription factor genes that were induced in mycorrhizal roots: AP2/ERF transcription factor required for mycorrhization 1 (*ERM1*, Medtr6g012970) and *ERF12* (Medtr2g014300)^7,16^ (Supplementary Table 1). ERM1 and ERF12 are specifically localised to the nucleus in *N. benthamiana* leaves and *Medicago* hairy roots (Supplementary Fig. 6). A Y1H assay using 250-bp *STR* and *STR2* promoters as baits showed that ERM1 bound strongly to AM-inducible *STR* and *STR2* promoter fragments, whereas WRI5a bound only to the *STR* promoter (Fig. 2c). A dual-luciferase reporter (DLR) assay revealed that ERM1 significantly transactivated the expression of *STR* and *STR2* in *N. benthamiana* leaves, and deletion of both AW-box/AW-box-like motifs eliminated this transactivation activity (Fig. 2d, e)^16^. Similar to the overexpression of *WRI5a*, overexpression of *ERM1* in *M. truncatula* hairy roots activated the expression of genes related to lipid transport (*STR* and *STR2*) and biosynthesis (*PK*, *KAS II*, *KAR*, *FatM*, and *RAM2*) under non-mycorrhized conditions (Supplementary Fig. 7)^16^.

Chromatin immunoprecipitation (ChIP) was performed using *M. truncatula* hairy roots transformed with ERM1-FLAG, WRI5a-FLAG, and GFP-FLAG (negative control), and qPCR primers were designed to amplify the AW-box/AW-like box regions of the *STR* and *STR2* promoters (Fig. 2f). Consistent with the Y1H results, the ChIP-qPCR results revealed that ERM1 was associated with the *STR* and *STR2* promoter fragments that contained AW-boxes (A1 site −97/−259) and AW-like boxes (A2 site −489/−659; B1 site −100/−200), whereas WRI5a was possibly associated with the AW-boxes in the *STR* promoter (Fig. 2c, f). Together, these data demonstrated that ERM1 functions as a transcriptional activator that regulates lipid metabolism and transport by binding directly to promoters of downstream genes such as *STR* and *STR2* via AW-box and AW-box-like motifs, thereby activating their expression.

### ERM1 is required for arbuscule formation

**Fig. 3:**
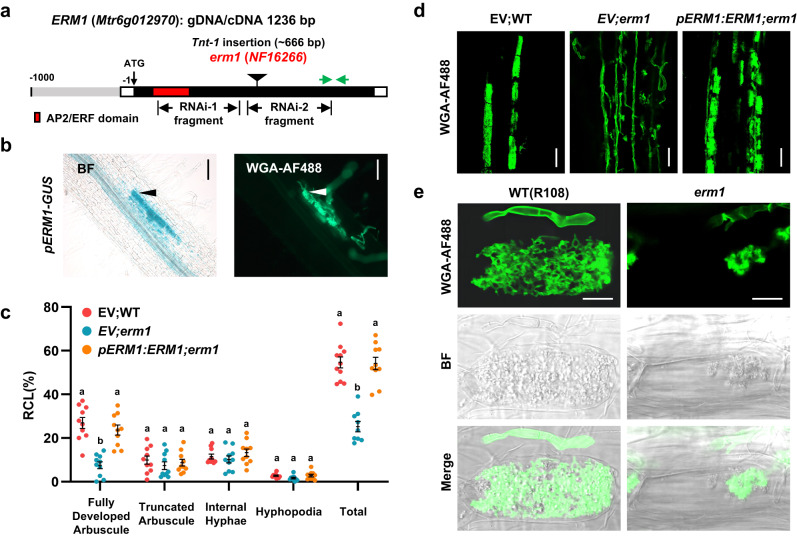
*ERM1* is required for arbuscule development. **a** Schematic of the *erm1* mutant (*NF16266*). The *Tnt1* insertion site is indicated by a triangle. The grey, black, and white boxes indicate the promoter, exon, and 5′/3′-UTR regions, respectively. Green arrows correspond to primers used to specifically quantify the *ERM1* expression level. One AP2/ERF domain is labelled in red. **b** Localisation of *ERM1* expression assessed using 1-kb promoter–GUS fusions in mycorrhized *M. truncatula* roots at 4 wpi. BF: Bright-field for GUS staining; WGA-AF488: fluorescence microscopy for fungal structures. Arrowheads indicate cells containing arbuscules. Scale bar, 50 μm. Experiments were repeated 3 times with similar results. **c**–**d** Quantification of *R. irregularis* colonisation level (**c**) and images of WGA-AF488-stained arbuscules (**d**) in WT (R108), *erm1* (*NF16266*), and *pERM1:ERM1;erm1* (*erm1* transformed with *ERM1* cDNA driven by its native promoter) at 6 wpi. EV, empty vector. Scale bar, 50 μm. **e** WGA-AF488-stained arbuscule phenotypes in R108 and *erm1* root cortical cells. Scale bar, 10 μm. Statistics: Individual data points and mean ± SE are shown in (**c**). Different letters indicate significant differences (One-way ANOVA, Duncan’s multiple range test, *P* < 0.01). Exact *P* values are provided in Source Data. *n* = 10 biologically independent plants.

To examine the function of *ERM1* in arbuscule development, we constructed a 1-kb *ERM1* promoter fragment driving GUS. We found that p*ERM1*:*GUS* was specifically expressed in arbuscule-containing cells during the mycorrhizal symbiosis (Fig. 3a, b and Supplementary Fig. 8a). We also identified an *erm1* mutant (*NF16266*) that contains a *Tnt*−1 insertion within the *ERM1* genomic sequence located 666 bp downstream of the start codon in the *ERM1* cDNA (Fig. 3a and Supplementary Fig. 9a, b). The *erm1* mutant showed significant defects in arbuscule development and lower expression of the AM-specific gene *PT4* compared with the wild type (R108) at 6 wpi with *R. irregularis*, although a few smaller arbuscules were observed (Fig. 3c–e and Supplementary Fig. 9b, c). Mycorrhizal colonisation and arbuscule development, together with *PT4* expression level in the *erm1* mutant, were restored to wild-type levels when *erm1* was complemented with *ERM1* driven by its native 1-kb promoter (Fig. 3c, d and Supplementary Fig. 9b, c).

We also investigated the role of *ERM1* using RNAi-mediated knockdown assays (Fig. 3a and Supplementary Fig. 10). *ERM1* expression levels were significantly reduced in two independent RNAi composite *M. truncatula* roots, *ERM1* RNAi-1 and RNAi-2 (Fig. 3a and Supplementary Fig. 10a). Similar to the phenotypes of *erm1* plants, the *ERM1*-RNAi plants exhibited smaller arbuscules and lower AM-specific induction of *STR/STR2* and *PT4* than plants transformed with the empty vector (EV) (Supplementary Fig. 10b–e).

Consistent with activation of lipid biosynthesis gene expression in *M. truncatula* hairy roots, *ERM1* overexpression (*35* *S:ERM1*) resulted in an increase in FA content and an enhanced arbuscule abundance after inoculation with *R. irregularis* (Supplementary Fig. 7, 11, 12). Collectively, these data suggest that *ERM1* is required for arbuscule formation and is likely to be involved in host lipid provision during the mycorrhizal symbiosis.

### ERF12 is activated by ERM1/WRI5a and negatively regulates arbuscule development

**Fig. 4:**
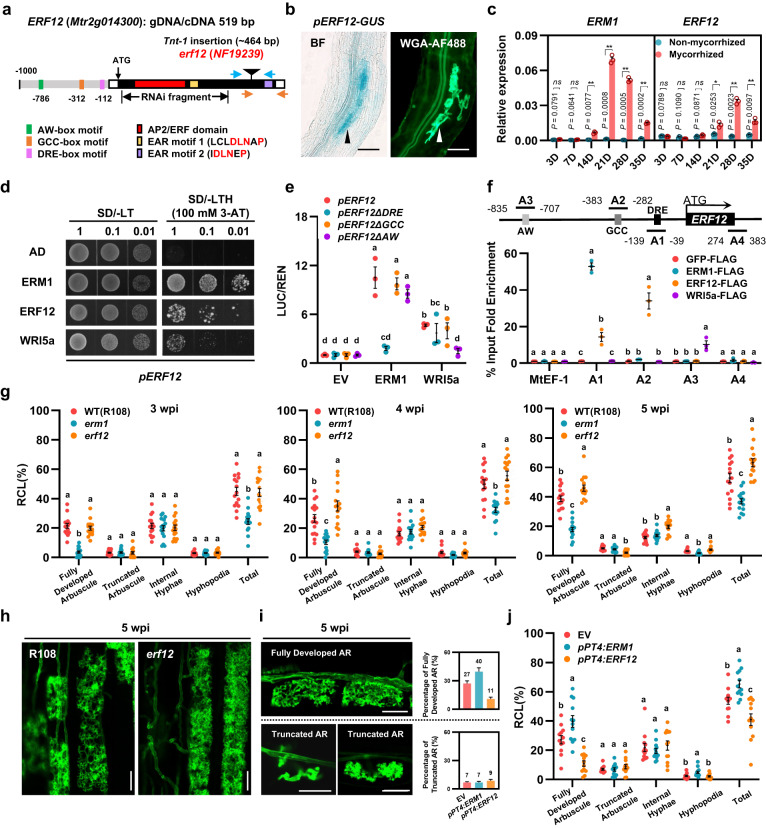
*ERF12* is activated by ERM1/WRI5a and negatively regulates arbuscule development. **a** Schematic representation of the *erf12* mutant (*NF19239*). The *Tnt1* insertion site is indicated by a triangle. The grey, black, and white boxes indicate the promoter, exon, and 5′/3′-UTR regions, respectively. The key domains (including one AP2/ERF-domain and two EAR repression motifs) and *cis*-elements (AW-box, GCC-box, and DRE-box) are labelled with different coloured boxes. **b** Localisation of *ERF12* expression assessed using 1-kb promoter–GUS fusions in mycorrhized *M. truncatula* roots at 4 wpi. Arrowheads indicate cells containing arbuscules. Scale bar, 100 μm. Experiments were repeated 3 times with similar results. **c** Relative expression levels of *ERM1* and *ERF12* at 3, 7, 14, 21, 28, and 35 days (D) after inoculation with *R. irregularis*. Relative expression was normalised to that of *MtEF-1*. **d** ERM1, ERF12, and WRI5a interact with the 1000-bp *ERF12* promoter in a Y1H assay. The numbers on the top of each photograph indicate the different concentrations of yeast cells. 3-AT, 3-amino-1,2,4-triazole. **e** In vivo transcriptional activation of the *ERF12* promoter with various deletions of indicated elements (ΔDRE/ΔGCC/ΔAW) by ERM1 or WRI5a assessed using a dual-luciferase system. **f** Anti-FLAG ChIP-qPCR analysis of ERM1, ERF12, and WRI5a binding to the *ERF12* promoter. **g** Quantification of *R. irregularis* colonisation level for WT (R108), *erm1*, and *erf12* at 3, 4, and 5 wpi. **h** WGA-AF488-stained arbuscule phenotypes in R108 and *erf12* root cortical cells at 5 wpi. Scale bar, 20 μm. **i** Representative images of WGA-AF488-stained fully developed and truncated arbuscules in *M. truncatula* hairy roots (A17) transformed with EV, *pPT4:ERM1*, and *pPT4:ERF12* at 5 wpi. The numbers on the right bar chart indicate the colonisation rate (%) of the two arbuscule types for the indicated genotypes. The individual data points and statistical analysis are shown in Fig. 4j. Scale bar, 20 μm. AR, arbuscule. **j** Quantification of *R. irregularis* colonisation level in *M. truncatula* hairy roots (A17) for EV, *pPT4:ERM1*, and *pPT4:ERF12* at 5 wpi. Statistics: Individual data points and mean ± SE are shown. **c** Two-sided Student’s *t* test. *P < 0.05; **P < 0.01; *ns* not significant. *n* = 3 technical replicates. Different letters (**e**–**g**, **j**) indicate significant differences (One-way ANOVA, Duncan’s multiple range test, *P* < 0.05). Exact *P* values are provided in Source Data. **e**–**f** *n* = 3 biologically independent samples; **g** *n* = 15 biologically independent plants; **j** *n* = 12 biologically independent plants.

Phylogenetic analysis showed that ERF12 and ERM1 are in the adjacent evolutionary clade belonging to the ERF family of the AP2/ERF superfamily; each contains a single AP2 domain and has no introns in its genomic sequence^36^ (Fig. 4a and Supplementary Fig. 13, 14). A 1-kb *ERF12* promoter fragment was sufficient to drive *GUS* expression specifically in arbuscule-containing cells after inoculation with AM fungi (Fig. 4b and Supplementary Fig. 8b). However, Y1H assay results indicated that ERF12 did not bind to the *STR/STR2* promoters (Fig. 2c), and transactivation assays revealed that ERF12 did not directly promote activation of *STR/STR2* in *M. truncatula* hairy roots or *N. benthamiana* leaves (Supplementary Fig. 15).

Induction of *ERF12* by AM occurred after that of *ERM1* and *WRI5a*: significant induction of *ERM1* and *WRI5a* began at 14 days post-inoculation (dpi) with *R. irregularis*, whereas that of *ERF12* began at 21 dpi (Fig. 4c and Supplementary Fig. 16). AW-box (−786/−799), GCC-box (AGCCGCC) (−312/−318), and DRE-box (A/GCCGAC) (−112/−117) *cis*-elements were found in the 1-kb *ERF12* promoter, suggesting that ERM1 and WRI5a might directly regulate *ERF12* expression during the AM symbiosis^16,17,37–39^ (Fig. 4a). Consistent with this hypothesis, Y1H, transactivation, and ChIP-qPCR assays revealed that ERM1 directly and strongly targeted an *ERF12* promoter fragment containing a DRE-box (A1 site −39/−139) to activate transcription, whereas WRI5a bound to an *ERF12* promoter fragment containing an AW-box motif (A3 site −707/−835) and transactivated *ERF12* expression (Fig. 4d−f and Supplementary Fig. 7, 17).

We noted that the *erf12* mutant (with a *Tnt*−1 insertion 464 bp downstream of the start codon in the *ERF12* cDNA) showed a significant increase in the abundance of fully developed arbuscules and higher AM-specific *PT4* induction at 6 wpi with *R. irregularis* (Fig. 4a and Supplementary Fig. 18). When *erf12* was complemented by *ERF12* driven by its native promoter, the AM colonisation level of *erf12* returned to a level similar to that of wild-type R108 (Supplementary Fig. 18).

To further examine the effects of *ERF12* on the AM symbiosis, we performed a time-course experiment to evaluate the mycorrhizal phenotypes of *erf12* and *erm1* at 3, 4, and 5 wpi. At 3 wpi with *R. irregularis*, the colonisation levels of *erf12* and the wild type (R108) were comparable, whereas *erm1* showed significant defects in arbuscule development compared with the wild type. However, the abundance of fully developed arbuscules became significantly higher in *erf12* than in the wild type at 4 wpi, and this difference was even greater at 5 wpi, consistent with specific induction of *ERF12* by AM in the later stage of the symbiosis (Fig. 4c, g, h and Supplementary Fig. 19). Transcript levels of *STR*, *STR2*, and *PT4* confirmed the visual phenotyping results (Supplementary Fig. 20).

We next overexpressed *ERF12* (p*PT4:ERF12*) and *ERM1* (p*PT4:ERM1*) in *M. truncatula* hairy roots using an arbuscule-specific *PT4* promoter. Compared with EV-transformed roots, p*PT4:ERF12* roots had stunted arbuscules and lower colonisation levels, whereas p*PT4:ERM1* roots exhibited significantly more arbuscules (Fig. 4i, j and Supplementary Fig. 21). Cutin content was significantly higher in *erf12* than in R108 but significantly lower in *erm1* (Supplementary Fig. 22).

The above data suggest that *ERF12* is activated by ERM1/WRI5a and negatively regulates arbuscule development in the later stage of the AM symbiosis, probably by affecting lipid transport.

### MtTPR3a interacts with ERF12 and negatively regulates arbuscule development

Two conserved EAR (ERF Amphiphilic Repression) motifs (defined as (L/F)DLN(L/F)xP, including the LxLxL, DLNxP, and DLNxxP variants) were identified in ERF12; one was adjacent to the AP2 domain (LCLDLNAP, EAR motif 1), and the other was at the C terminus (IDLNEP, EAR motif 2; Fig. 4a). In *Arabidopsis*, several AP2/ERF family genes with EAR motifs act as transcriptional repressors that regulate plant responses to developmental cues, hormone signalling, and abiotic and biotic stress^38–43^.

**Fig. 5:**
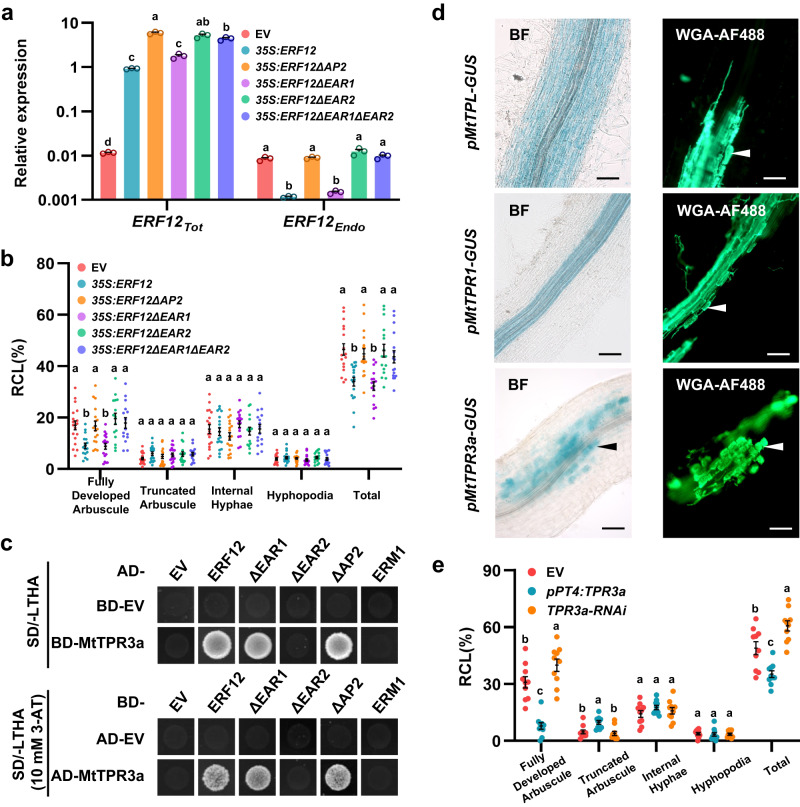
MtTPR3a interacts with ERF12 and negatively regulates arbuscule development. **a** Relative gene expression of overexpressed *ERF12* transcripts (*ERF12*_*Tot*_) and full-length *ERF12* mRNA (*ERF12*_*Endo*_) in non-mycorrhized *M. truncatula* hairy roots (A17) transformed with the EV, *ERF12*-overexpression construct (*35* *S:ERF12*), and *ERF12-*overexpression construct without the AP2 domain (*35* *S:ERF12ΔAP2*) or single/double EAR motifs (*35* *S:ERF12-ΔEAR1/ΔEAR2/ΔEAR1ΔEAR2*). Relative expression was normalised to that of *MtEF-1*. The blue and orange arrows in Fig. 4a indicate the primer positions used to measure mRNA abundance of *ERF12*_*Tot*_ and *ERF12*_*Endo*_. **b** Quantification of *R. irregularis* colonisation level in plant roots corresponding to (**a**) at 5 wpi. **c** The C-terminal EAR motif 2 of ERF12 is required for interaction with MtTPR3a in a yeast two-hybrid (Y2H) assay. Full-length (ERF12) and domain-deleted ERF12 protein fragments (ΔEAR1, ΔEAR2, and ΔAP2) were used as prey or baits with MtTPR3a used as a bait or prey, respectively. **d** Bright-field (left) and corresponding fluorescence microscopy (right) images for localisation of *MtTPL*, *MtTPR1*, and *MtTPR3a* expression, which was assessed using 1-, 2.1-, and 1.6-kb promoter–GUS fusions in mycorrhized *M. truncatula* roots (A17), respectively. Arrowheads indicate cells containing arbuscules. Scale bar, 50 μm. Experiments were repeated twice with similar results. **e** Quantification of *R. irregularis* colonisation level in *M. truncatula* hairy roots for EV (A17), *pPT4:TPR3a*, and *TPR3a-RNAi* at 5 wpi. Statistics: Individual data points and mean ± SE are shown. Different letters (**a**, **b**, **e)** indicate significant differences (One-way ANOVA, Duncan’s multiple range test). Exact *P* values are provided in Source Data. **a** *n* = 3 technical replicates, *P* < 0.01; **b** *n* = 15 biologically independent plants, *P* < 0.01; **e** *n* = 10 biologically independent plants, *P* < 0.05.

To investigate the function of the EAR motifs in ERF12, we initially overexpressed *ERF12* in *M. truncatula* hairy roots and detected expression of endogenous *ERF12* (*ERF12 Endo*) using specific *ERF12* 3′-UTR primers (Fig. 4a). *ERF12 Endo* was strongly downregulated in *ERF12*-overexpressing (*35S:ERF12*) roots (Fig. 5a). Similar to p*PT4:ERF12* roots (Fig. 4j), *35S:ERF12* plants showed significantly compromised arbuscule development at 5 wpi with *R. irregularis* compared with the EV control (Fig. 5b). When the AP2 domain (*35S:ERF12ΔAP2*) and the single and double EAR motifs (*35S:ERF12-ΔEAR1/ΔEAR2/ΔEAR1ΔEAR2*) of ERF12 were deleted, *ERF12 Endo* expression levels and AM colonisation in *35S:ERF12ΔAP2*, *35S:ERF12ΔEAR2*, and *35S:ERF12ΔEAR1ΔEAR2* hairy roots were similar to those of EV roots. These results indicated that the AP2 domain and C-terminal EAR motif 2 were required for the negative function of ERF12 in arbuscule development (Fig. 5a, b). Y1H and ChIP-qPCR assays revealed that ERF12 bound directly to its own promoter fragments that contained a GCC-box (A2 site −282/−383) and a DRE-box (A1 site −39/−139) (Fig. 4d, f). Therefore, ERF12 associates with its own promoter and suppresses its own expression through C-terminal EAR motif–mediated transcriptional repression.

Because EAR-containing proteins can induce transcriptional repression via the chromatin-remodelling machinery by recruiting TPL/TPR (TOPLESS/TOPLESS-related) co-repressors^39–45^, we next asked whether TPL/TPR co-repressors participated in ERF12-mediated transcriptional inhibition during the mycorrhizal symbiosis. Nine predicted TPL/TPR-like proteins were identified in *Medicago* (Supplementary Fig. 23). Expression of *MtTPL*, *MtTPR1*, *MtTPR2*, *MtTPR3a*, *MtTPR3b*, and *MtTPR4* was detected in *M. truncatula* roots, and these genes were used for further analysis (Supplementary Fig. 24 and Supplementary Table 1). Yeast two-hybrid (Y2H) assays showed that MtTPL, MtTPR1, and MtTPR3a could interact with ERF12 (Fig. 5c and Supplementary Fig. 25). Because *MtTPR3a* was specifically expressed in arbuscule-containing cells (Fig. 5d, Supplementary Fig. 26, and Supplementary Table 1), we further investigated its role through *PT4* promoter-driven overexpression (p*PT4:TPR3a*) and RNAi-mediated knockdown assays (*TPR3a-*RNAi). p*PT4:TPR3a* roots showed a significant reduction in *R. irregularis* colonisation compared with EV-transformed hairy roots, whereas *TPR3a-*RNAi plants showed significantly more fully developed arbuscules at 6 wpi with *R. irregularis* (Fig. 5e and Supplementary Fig. 27). Y2H assays revealed that deletion of the C-terminal EAR motif abolished the interaction between ERF12 and MtTPR3a (Fig. 5c). We concluded that ERF12 interacts with MtTPR3a through the C-terminal EAR motif and forms a transcriptional repressor complex that negatively regulates arbuscule development.

### ERF12 interacts with and antagonises ERM1/WRI5a during arbuscule development

**Fig. 6:**
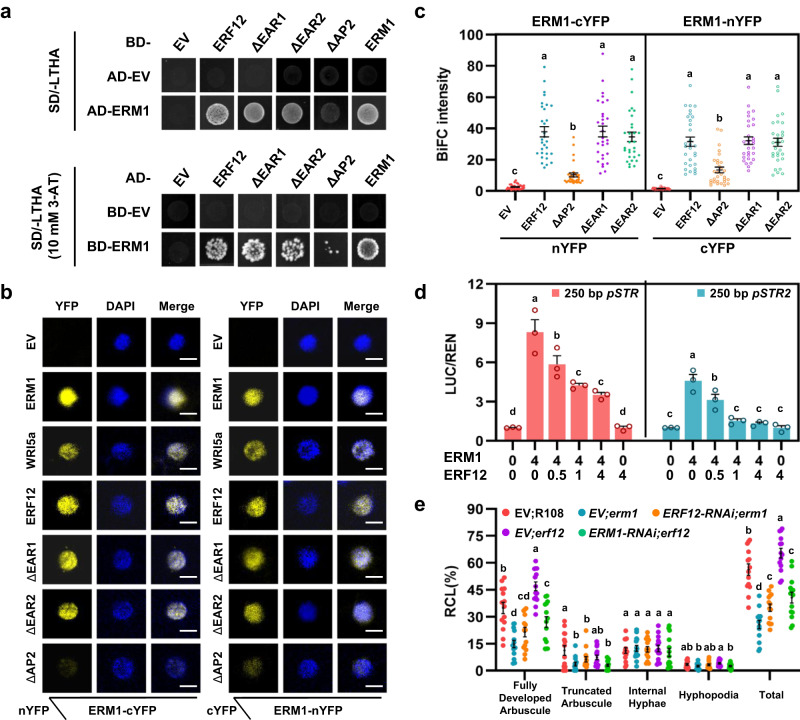
ERF12 interacts with and antagonises ERM1/WRI5a during arbuscule development. **a** The AP2 domain of ERF12 is required for interaction with ERM1 in a Y2H assay. Full-length (ERF12) and domain-deleted ERF12 protein fragments (ΔEAR1, ΔEAR2, and ΔAP2) were used as prey and baits with ERM1 used as a bait and prey, respectively. **b** BiFC assay showing protein–protein interactions in *N. benthamiana* leaves. YFP fluorescence signals were detected in leaves co-transformed with the indicated BiFC combinations. ΔEAR1, ΔEAR2, and ΔAP2 indicate ERF12 protein fragments in which the corresponding domain has been deleted. nYFP, N-terminal fragment of YFP; cYFP, C-terminal fragment of YFP; DAPI, 4ʹ,6-diamidino-2-phenylindole for nuclear staining; Merge, YFP + DAPI. Scale bar, 5 μm. **c** Quantification of YFP signal intensity (Leica Application Suite X 3.3.0 software) from BiFC combinations of ERM1 and ERF12 with various deletions of the indicated domains corresponding to (**b**). **d** In vivo transcriptional activation of 250-bp *STR* and *STR2* promoters by an ERM1 and ERF12 effector mixture assessed with a dual-luciferase system. Transgenic *Agrobacteria* expressing ERM1 and ERF12 were mixed at different concentrations for infiltration. **e** Quantification of *R. irregularis* colonisation level in *M. truncatula* hairy roots expressing the EV or RNAi targeting *ERF12* in the *erm1* background (EV;*erm1* and *ERF12-RNAi;erm1*, respectively), EV or RNAi targeting *ERM1* in the *erf12* background (EV;*erf12* and *ERM1-RNAi/erf12*, respectively), and the EV control in the WT (R108) background (EV;WT) at 6 wpi. Statistics: Individual data points and mean ± SE are shown. Different letters (**c**–**e**) indicate significant differences (One-way ANOVA, Duncan’s multiple range test, *P* < 0.05). Exact *P* values are provided in Source Data. **c** *n* = 30 cells for each BiFC combination; **d** *n* = 3 biological replicates; **e** *n* = 14 biologically independent plants.

EAR repressors act either by interacting with co-repressors and binding to promoters or by directly/indirectly modulating the functional status of transcriptional activators^38,41,42,44^. To further investigate the interplay between the transcriptional activators ERM1/WRI5a and the repressor ERF12, we assessed whether these three members might form negative feedback loops to fine-tune arbuscule development. Y2H assays and BiFC assays in *N. benthamiana* leaves showed that the three proteins could physically interact with one another, and the AP2 domain in ERF12 was required for its interaction with ERM1 and WRI5a (Fig. 6a–c and Supplementary Figs. 28, 29). Therefore, these proteins can form homo- and/or heterodimer complexes, and ERF12 may affect the function of ERM1/WRI5a through protein–protein interaction.

We found that ERF12 did not affect transcript levels of *ERM1* or *WRI5a* in *35S:ERF12 M. truncatula* hairy roots (Supplementary Fig. 15a), and we therefore hypothesised that the ERF12 repressor might functionally interact with transcriptional activators to curtail its activity. This hypothesis was supported by the results of a transactivation assay in *N. benthamiana* leaves: ERM1 significantly enhanced luciferase activity driven by the 250-bp *STR* and *STR2* promoters. However, as the amount of co-transfected ERF12 increased, luciferase activity gradually decreased to basal levels (Fig. 6d). Similar results were obtained when we co-transfected ERF12 and WRI5a (Supplementary Fig. 30). Intriguingly, we found that transactivation of the *ERF12* promoter mediated by ERM1/WRI5a could be inhibited by ERF12 itself (Supplementary Fig. 31). Therefore, ERM1/WRI5a-induced transactivation of related target genes (*STR*, *STR2*, and *ERF12*) was competitively inhibited by ERF12 because of the interaction between ERF12 and ERM1/WRI5a.

To further study the genetic relationship between the transcriptional activator ERM1 and the repressor ERF12, we performed RNAi targeting of *ERF12* in the *erm1* background (*ERF12-RNAi;erm1*) and *ERM1* in the *erf12* background (*ERM1-RNAi;erf12*) by transforming *M. truncatula* hairy roots (Supplementary Fig. 32). *ERF12-RNAi;erm1* partially complemented the reduced AM colonisation of *EV;erm1*, whereas *ERM1-RNAi;erf12* exhibited less AM colonisation than *EV;erf12* at 6 wpi with *R. irregularis* (Fig. 6e). These results further support our observation that ERF12 suppresses the transcription of target genes by interacting with ERM1/WRI5a during the mycorrhizal symbiosis.

## Discussion

AM fungi require fatty acids synthesised in host plants to complete their life cycle^6,7,16,46^. Upon addition of exogenous fatty acids such as palmitoleic acid and myristate, AM fungi can grow without plants in culture medium and form infection-competent secondary spores, suggesting that dependence on host lipids is the basis for the obligate symbiosis of AM fungi^47,48^. The AP2-domain transcription factors WRI5a and CBX1 regulate bidirectional lipid and phosphate exchange in both *M. truncatula* and *L. japonicus*, further suggesting that the plant–AM fungus mutualistic interaction is regulated mainly by the plant host^16,17^. In this study, we showed that ERM1 and WRI5a formed heterodimers and directly activated the expression of *STR* via the AW-box motif; we also demonstrated that ERM1 activated the expression of *STR/STR2* via the AW-box-like motif (Figs. 2, 6). The co-expression of *ERM1* and *WRI5a* in *N. benthamiana* leaves led to further *STR* and *STR2* transactivation (although this difference was not significant), suggesting that ERM1 and WRI5a may have a synergistic effect on the activation of target genes (Supplementary Fig. 33). Because the AP2-domain transcription factors WRI5a, WRI5b/Erf1, and WRI5c may function redundantly in the AM symbiosis^16^, we constructed double mutants of *ERM1* and *WRI5a* by RNAi transformation of *M. truncatula* hairy roots. The compromised arbuscule development in the two independent *ERM1-WRI5a*-double RNAi plants was similar to that of *ERM1*-single RNAi plants, further suggesting that ERM1 and WRI5a may regulate mycorrhizal symbiosis through dimer formation (Supplementary Fig. 34). OsPHR2 binds directly to the promoters of *OsRAM1*, *OsWRI5A*, and *OsPT11* via P1BS (GnATATnC) *cis*-elements and activates AM-specific genes^18,28,49,50^. However, given the absence of a P1BS motif in the 1-kb promoter of *ERM1*, the relationships among PHRs, RAM1, ERM1, and WRI5a should be investigated further (Supplementary Fig. 35).

Our previous study indicated that *RAM1* and *WRI5a* positively regulate each other^16^. Similarly, we found that RAM1 could transactivate the promoter of *ERM1* in *N. benthamiana* leaves and enhance its expression in *M. truncatula* hairy roots (Supplementary Fig. 36). Overexpression of *ERM1* in *M. truncatula* hairy roots also led to activation of *WRI5a, RAM1*, and *PT4*, as well as genes related to lipid biosynthesis and transport (Supplementary Fig. 7), suggesting that *RAM1*, *ERM1*, and *WRI5a* might regulate one another to form a positive feedback loop. Further analysis showed that ERM1 was able to transactivate the promoter of *PT4* in an AW-box/AW-like box–dependent manner in *N. benthamiana* leaves, which was consistent with the presence of an AW-box motif and two AW-box-like motifs in its 1-kb promoter (Supplementary Figs. 35, 37). These results suggested that, like WRI5a, ERM1 might participate in the lipid–phosphate bidirectional nutrient exchange in *Medicago*^16^.

Half-size ABCG transporters must undergo homo- or heterodimerisation to form functional ABC transporters that translocate substrates across the membrane^51^. In the non-mycorrhizal species *Arabidopsis*, AtABCG11/WBC11 participates in a variety of biological processes by undergoing self-oligomerisation or forming heterodimers with several other ABCG transporters, which are necessary for plant development^52–54^. Both our study (Supplementary Fig. 1) and that of Zhang et al. showed that neither STR nor STR2 could homodimerise, but instead they formed heterodimers with each other^20^. Thus, STR/STR2 might perform a specific lipid efflux function during the AM symbiosis through dimer formation. This may be one of the reasons why *STR/STR2* co-overexpression only partially compensated for the lipid transport defects of *atwbc11-4*. However, we speculate that STR2 may also be involved in biological processes other than the AM symbiosis. Previous studies showed that *STR2* expression was higher than that of *STR* in non-mycorrhized conditions^7,16,20^ (Supplementary Fig. 5), and our *str2* mutants showed distinct developmental phenotypes such as dwarfing and reduced seed number and weight compared with R108 (Supplementary Fig. 38). Although AW-box-like motifs are necessary to specifically induce *STR2* in arbuscule-containing cells, the 250-bp *STR2* promoter fragment without the two AW-box-like motifs can still drive weak induction of *GUS* expression in infected cortex areas compared with non-symbiosis conditions (Fig. 2b and Supplementary Fig. 5). In addition, although WRI5a failed to bind to the 250-bp *STR2* promoter (Y1H assay) and fragments containing AW-like boxes (ChIP-qPCR assay), WRI5a significantly transactivated *STR2* in *N. benthamiana* leaves, independent of AW-like boxes (Fig. 2c, 2e–f). Thus, additional *cis*-elements and/or transcription factors may be involved in activating the *STR2* promoter. Further studies are needed to determine whether STR2 participates in plant development and the AM symbiosis by forming different dimers to transport distinct substrates.

Negative regulatory mechanisms have evolved to ensure the fine-tuning of different stages of the AM symbiosis and to maintain its symbiotic nature. For instance, SPX proteins function as phosphate sensors and specifically suppress PHR2-mediated transcriptional activation of AM-related genes at high plant phosphate status; consequently, the AM symbiosis is inhibited in order to maintain a beneficial nutrient exchange for plants^18,28,29,49,50^. The AM-inducible MYB-like transcription factor MYB1 forms a transcription factor complex with DELLA and NSP1 to promote arbuscule degeneration^55^. In the present study, the *Medicago* transcriptional repressor MtERF12, activated by MtERM1/WRI5a upon mycorrhizal symbiosis, could mediate its own feedback inhibition through its AP2 DNA-binding domain and recruit TOPLESS-MtTPR3 to negatively regulate arbuscule development. This process might contribute to maintaining the homoeostasis of bidirectional nutrient exchange during the AM symbiosis (Figs. 4–6). Instead of binding to the promoter of common downstream target genes, such as *STR/STR2*, to directly suppress their activation, MtERF12 forms homo- and/or heterodimer complexes with MtERM1 and WRI5a to curtail their activity (Figs. 2c, 6a–d, and Supplementary Fig. 28–30). MtERF12 interacted with MtERM1/WRI5a through the AP2 domain and with its co-repressor MtTPR3a, an AM-inducible TPL/TPR member, through the C-terminal EAR motif (Figs. 5c, 6a–c, and Supplementary Figs. 25, 28, 29). This result indicated that ERF12 acts as a bridge to connect ERM1/WRI5a with the TPL/TPR co-repressor, thereby converting them to transcriptional repressors. A recent study demonstrated that the EAR-containing SlERF.F12 in *Solanum lycopersicum* suppresses fruit ripening by recruiting the SlTPL2 co-repressor and the chromatin modifier proteins HDA1/HDA3, thereby epigenetically suppressing expression of ripening-related genes in tomato^45^. However, assays should be performed to determine whether chromatin-remodelling factors are recruited by MtERF12 to epigenetically suppress gene expression in the mycorrhizal symbiosis.

**Fig. 7:**
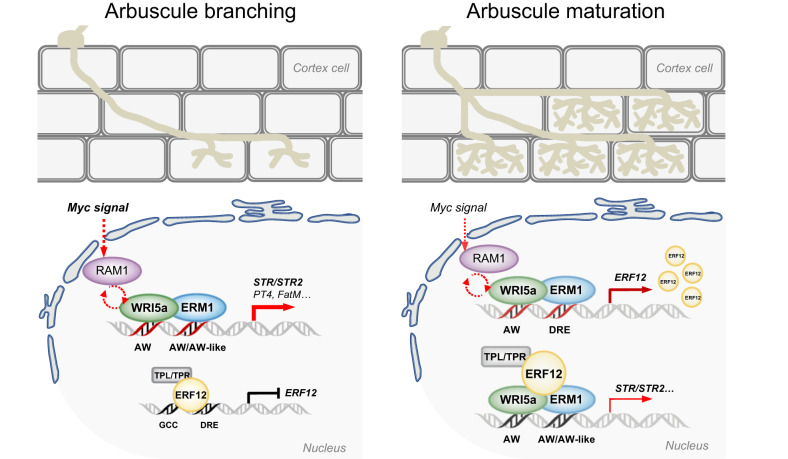
Proposed model of the ERM1/WRI5a–ERF12–TOPLESS module in the mycorrhizal symbiosis. At the early stage of arbuscule development, the *Medicago* AP2/ERF transcriptional activators ERM1 and WRI5a are activated downstream of RAM1 to increase gene expression by directly binding to AW-box/AW-box-like *cis*-elements in the *STR* and *STR2* promoters, thus quickly rewarding AM fungi. *ERF12* is negatively autoregulated by binding to the GCC-box and DRE-box in its own promoter and recruiting the TPL/TPR co-repressor. With increasing arbuscule abundance, ERF12 protein accumulates under the coordinated regulation of ERM1 and WRI5a. In turn, ERF12 interacts with ERM1 and WRI5a to negatively regulate its transcriptional activation; it also likely recruits TPL/TPR co-repressors such as MtTPR3a to actively suppress their transactivation activity, thus inhibiting STR/STR2-mediated arbuscular lipid provision. The self-inhibition of ERF12 leads to a gradual decrease in its expression level; when a threshold value is reached, it can be activated again. This strictly regulated but flexible ERM1/WRI5a–ERF12–TOPLESS feedback module ensures the stability of the AM symbiosis.

In summary, our results support a model in which a transcriptional negative feedback loop coordinates arbuscule development and symbiotic nutrient exchange (Fig. 7). At the early stage of arbuscule development, symbiotically activated RAM1-WRI5a/ERM1 can reprogram root cortex cells as required for arbuscule development and nutrient exchange^7,22^. ERM1 and WRI5a act as transcriptional activators to activate the expression of genes involved in fatty acid biosynthesis and transfer, including *FatM*, *STR*, and *STR2*, by binding directly to their promoters^7,16^. Concomitantly, ERM1 and WRI5a activate *MtERF12*. ERF12 then interacts with MtERM1/WRI5a and recruits the TOPLESS co-repressor to counteract MtERM1/WRI5a function, thereby suppressing lipid biosynthesis and transfer. These findings suggest that the regulatory mechanism of nutrient exchange is not an on/off process; instead, its intensity and spatiotemporal activity are modulated by additional players. This mechanism promotes appropriate resource allocation according to plant growth and development to maintain the balance of symbiotic nutrient exchange. Thus, the dose-dependent transcriptional regulatory complex of ERM1/WRI5a–ERF12–TOPLESS performs ‘activation-inhibition’ dynamic regulation in arbuscule-containing cells, enabling the maintenance of a stable, reciprocally beneficial symbiosis.

## Methods

### *M. truncatula* materials, hairy root transformation, and mycorrhizal infection

The *Rhizophagus irregularis* (syn. *Glomus intraradices*) inoculum and *M. truncatula wri5a* (*NF13926*) *Tnt1* mutants used in this study have been described^6,16^. The *M. truncatula STR2* (Medtr5g030910) mutant *str2* (*NF11835*), *ERM1* (Medtr6g012970) mutant *erm1* (*NF16266*), and *ERF12* (Medtr2g014300) mutant *erf12* (*NF19239*) were obtained from the *Medicago truncatula* Mutant Database (https://medicago-mutant.dasnr.okstate.edu/mutant/index.php). The *str2*, *erm1*, *erf12*, and *wri5a* backgrounds were usually R108. The genotypes of the *Tnt1* mutants were confirmed by PCR using the transposon-specific primer Tnt1-F2 together with the corresponding gene-specific primers listed in Supplementary Table 2.

*M. truncatula* chimeric transgenic plants were obtained by hairy root transformation as described by Boisson-Dernier et al.^56^, and mycorrhizal inoculum was prepared using the method of Jiang et al.^6^. In brief, seeds were germinated on 1% water agar plates after scarification with H_2_SO_4_ and surface sterilisation with 10% (v/v) bleach. *Agrobacterium rhizogenes* strain *Arqua*−1 containing the target constructs was used for *M. truncatula* hairy root transformation. Four weeks later, transformed composite plants with fluorescent hairy roots (pK7WG2R, pK7GWIWGIIR, and pK7WG2Rδ35S vector with dsRed tag) were transferred to a greenhouse (22 °C with a 16-h light/8-h dark photoperiod) and grown in a 1:1 mixture of perlite and sand inoculated with ~400 *R. irregularis* spores per plant. After culture for 3–6 weeks (according to the specific study purposes), plants with uniform fluorescence intensity and growth were selected for measurement of AM colonisation rate. The arbuscules were divided into two types according to their size relative to cortical cells, as shown in Fig. 4i and Supplementary Fig. 2f. Fully developed arbuscules were defined as arbuscules with a square shape that filled the cortical cell, whereas truncated arbuscules were defined as those that were collapsed, wrinkled, and did not fill the whole cell. For ‘nurse plant’ experiments, wild-type and mutant seedlings were planted together in the same pot separated by a 125-μm nylon mesh.

### *Arabidopsis* materials, growth conditions, and transformation

The *A. thaliana AtWBC11* (At1g17840) mutant *atwbc11-4* (*Salk_043637*) used in this study was a kind gift of Dr. Xiaoya Cheng^34^. Because homozygous *atwbc11-4* plants were sterile, the mutants were maintained in a heterozygous state. *A. thaliana* seeds were surface sterilised and sown on plates containing half-strength Murashige and Skoog (MS) medium. Ten days after germination, seedlings were transplanted into soil in a growth room at 22 °C with a long-day photoperiod (16-h light/8-h dark). *A. tumefaciens* GV3101 strains containing target constructs were used for *A*. *thaliana* transformation by the floral dip method^57^. Transgenic plants co-overexpressing *STR-STR2* (in the *atwbc11-4* background) were selected on half-strength MS medium containing 50 μg/mL kanamycin (Sigma-Aldrich), and T3 homozygous transgenic plants were used for phenotyping.

### Microscopy

Mycorrhized *M. truncatula* roots were washed and treated with 10% KOH for 6 min at 95 °C, then placed in ink/acetic acid/water (5:5:90, v/v/v) for 3 min as described by Vierheilig et al.^58^. *R. irregularis* colonisation level was quantified using the grid-line intersect method as described by Giovannetti and Mosse^59^ and imaged under an Olympus MVX10 fluorescence microscope. Mycorrhized roots were also stained with WGA-Alexa Fluor 488^6,16^. In brief, harvested roots were placed in 50% ethanol for at least 4 h and then transferred to 20% (w/v) KOH for 2–3 days, followed by 0.1 M HCl for 1–2 h at room temperature. After HCl was removed, the sample was rinsed twice with distilled H_2_O and once with PBS buffer (pH 7.4), then immersed in PBS/WGA-Alexa Fluor 488 staining solution (0.2 μg/mL) in the dark for more than 6 h. Images of WGA-AF488-stained arbuscules were obtained under a Leica SP8 confocal microscope (Germany). GUS staining patterns were observed and photographed with a Zeiss Axio Imager A2 light microscope (Germany).

### Gene expression analysis

Total RNA was extracted from root tissues using TRIzol reagent (Invitrogen). First-strand cDNA was generated using the PrimeScript RT Reagent Kit with gDNA Eraser (TaKaRa). Quantitative Reverse Transcription PCR (qRT-PCR) was performed on a CFX Connect Real-Time System (BIO-RAD) using 2×RealStar Green Fast Mixture (GenStar). Relative expression was normalised to that of *M. truncatula Elongation factor 1* (*MtEF-1*) in *M. truncatula*^60^ and *PP2A* (At1g59830) in *A*. *thaliana*^61^. qRT-PCR conditions were as follows: 45 cycles of 95 °C for 15 s and 60 °C for 20 s. Primer sequences for qRT-PCR are listed in Supplementary Table 2.

### Plasmid construction

Genes and promoter regions were amplified with 2× Phanta Flash Master Mix (Vazyme, P520-01) from cDNA or genomic DNA using standard protocols and the primers listed in Supplementary Table 2. The PCR products were cloned into pENTR/SD/D-Topo (Invitrogen) and then transferred to the destination vector by Gateway LR reactions (Invitrogen) as indicated in Supplementary Table 3.

### Cutin monomer analysis, FAME extraction, and GC-QTOF-MS analysis

Cutin monomers and Fatty Acid Methyl Esters (FAMEs) were prepared and analysed by GC-QTOF-MS (gas chromatography with quadrupole time-of-flight mass spectrometry) using the method of Jiang et al.^6^. Data were acquired and evaluated with MassHunter Acquisition and MassHunter Quantitative and Qualitative Analysis (version B07, Agilent Technologies, CA, USA), respectively. Cutin monomers and FAMEs were identified by comparing their mass spectra with those in the standard solution (ANPEL, Shanghai, China) and the National Institute of Standards and Technology library (NIST 14). When calculating the mole% of fatty acids, we defined the sum of the most abundant fatty acid content from 16:0 to 24:0 fatty acid (16:0, 18:0, 18:1, 18:2, 20:0, 22:0, 24:0 fatty acid) as 100%.

### GUS histochemical staining

Positive transgenic lines harbouring the promoter–GUS reporter gene construct were stained in a solution comprised of 10.0 mM EDTA disodium salt, 0.1% [v/v] Triton X-100, 100 mM sodium phosphate buffer (pH 7.0), 0.5 mM potassium ferricyanide, 0.5 mM potassium ferrocyanide, and 0.5 mg/mL 5-bromo-4-chloro-3-indolyl-β-D-glucuronic acid at 37 °C for 0.5–2 h. The GUS staining reaction was terminated by washing with 75% ethanol.

### Yeast one-hybrid assay

To screen candidate transcription factors, 250 bp of the *STR2* promoter was amplified as the bait promoter and inserted into the pHIS2 vector. cDNA sequences of the AP2/ERF family proteins WRI5a, WRI5b, WRI5c, ERM1, and ERF12 were cloned into the pGADT7-GW AD vector. These vectors were co-transformed into the Y187 yeast strain (Clontech) as described in the user manual of the Matchmaker One-Hybrid Library (PT1031-1).

### Yeast two-hybrid assay

For Y2H assays, the target coding sequences of *ERM1*, *WRI5a*, *ERF12*, *MtTPL*, *MtTPR1*, *MtTPR2*, *MtTPR3a*, *MtTPR3b*, and *MtTPR4* were inserted into the pGADT7-GW AD and pGBKT7-GW BD vectors. To test for protein–protein interactions in yeast cells, different combinations of AD and BD vectors were co-transformed into the yeast strain AH109, and the clones were grown in synthetic dropout (SD) medium lacking leucine and tryptophan (SD/-LT) or leucine, tryptophan, histidine, and adenine (SD/-LTHA) with 3-amino-1,2,4-triazole (3-AT).

### BiFC assay

Overnight cultures of *A. tumefaciens* GV3101 strains harbouring the pXY106 and pXY104 recombinant plasmids were collected by centrifugation, resuspended in 2-[N-morpholino] ethanesulfonic acid (MES) buffer (10 mM MES [pH 5.6], 10 mM MgCl_2_, and 0.5 mM acetosyringone) to an OD_600_ of 2.0. Equal volumes of cells containing pXY106 and pXY104 were mixed and incubated at room temperature for 1 h before infiltration. An *A. tumefaciens* GV3101 suspension in a 1-mL needleless syringe was carefully press-infiltrated into healthy 4-week-old *N. benthamiana* leaves. Two days post-inoculation, fluorescent signals that suggested interactions between various protein pairs were detected and imaged using a Leica SP8 confocal microscope. The nucleus was stained with 10 μg/mL 4ʹ,6-diamidino-2-phenylindole (DAPI; Sangon Biotech, Shanghai, China) for 20 min. Quantification of the BiFC signal was performed using Leica Application Suite X (3.3.0) software.

### Transactivation assay (dual-luciferase system, DLR)

Effector plasmids (pGWB441 recombinant plasmids) and reporter plasmids (pGreenII-0800-LUC recombinant plasmids) were transformed into *A. tumefaciens* GV3101, and the pGreenII constructs were co-transformed with a pSoupP19 plasmid. The luciferase activity of the *N. benthamiana* extracts was analysed using a Dual-Luciferase Assay Kit (Promega) and detected on a Synergy 2 multimode microplate reader (Bio-Tek)^62^.

### Chromatin immunoprecipitation (ChIP) analysis

ChIP was performed as described by Saleh et al.^63^ and Fonouni-Farde et al.^64^ with some modifications. In brief, UBQ:ERM1-FLAG, UBQ:ERF12-FLAG, UBQ:WRI5a-FLAG, and UBQ:GFP-FLAG (negative control) constructs were separately transformed into *M. truncatula* hairy roots. The roots (6 weeks old) were harvested after *R. irregularis* infection, and DNA prepared from the roots was crosslinked with 30 mL of 1% formaldehyde/PBS buffer. After nuclei isolation, the crosslinked chromatin was sonicated using a Scientz18-A ultrasonic DNA interruptor (10 s on/15 s off for 50 cycles); the majority of the DNA fragments were between 200 and 1000 bp. The sheared chromatin was incubated with balanced anti-FLAG beads (Sigma) for 2 h and washed several times in different solutions according to the method described by Saleh et al.^63^. After elution of chromatin bound to the anti-FLAG beads, the samples were re-crosslinked by adding 5 M NaCl to the elution buffer and incubating at 65 °C overnight. The remaining steps for DNA purification were performed according to the manufacturer’s instructions, and immunoprecipitated DNA was resuspended in 25 μL of water. The immuno-precipitated DNA and total input DNA were analysed using ChIP-qPCR, and qPCR was performed on a real-time PCR detection system (Bio-Rad) with 2×RealStar Green Fast Mixture (GenStar, China). The PCR conditions used were: 45 cycles of 95 °C for 15 s, 60 °C for 15 s, and 72 °C for 15 s. The primers used are listed in Supplementary Table 2.

### Protein extraction and western blotting

For total protein extraction from yeast, a post-alkaline extraction method was performed. In brief, yeast cells were collected, resuspended in 100 μL 0.2 M NaOH (with 1 mM PMSF), incubated for 5 min at room temperature, pelleted and resuspended in 50 μL SDS loading buffer, and boiled for 5 min. The proteins were then separated using SDS–PAGE. Antibodies against the following proteins were used: GAL4-AD (Abbkine, ABP57231, 1:2000), GAL4-BD (Abbkine, ABP57232, 1:2000), and goat anti-rabbit IgG secondary antibody (Thermo Fisher, 31460, 1:10 000).

### Statistics

Data were analysed using Prism 8.0 Software (GraphPad, USA). Individual data points and mean ± SE are shown in the figures. Statistically significant differences between control and experimental groups were determined by one-way ANOVA (with Duncan’s multiple range test) or two-sided Student’s *t* test (*P < 0.05; **P < 0.01; *ns*, not significant). The *P*-value used in a given ANOVA analysis (*P* < 0.05 or *P* < 0.01) is indicated in the legends, and different letters indicate significant differences.

### Reporting summary

Further information on research design is available in the Nature Portfolio Reporting Summary linked to this article.

### Supplementary information

Supplementary Information

Reporting Summary

### Source data

Source data

## Data Availability

The data that support the findings of this study are available in the main text or supplementary information. The *M. truncatula Tnt1* transposon insertion lines used in this study were obtained from the *Medicago truncatula* Mutant Database (https://medicago-mutant.dasnr.okstate.edu/mutant/index.php). All materials are available from the corresponding author on request. Source data are provided with this paper.
