# Supplementary material for: Control of arbuscule development by a transcriptional negative feedback loop in Medicago
Source: Nat Commun. 2023 Sep 16;14:5743. doi: 10.1038/s41467-023-41493-2 (PMC10505183; doi:10.1038/s41467-023-41493-2)
Supplement: Supplementary file 1 — Supplementary Information [file 41467_2023_41493_MOESM1_ESM.pdf]

Supplementary information

Control of arbuscule development by a transcriptional negative feedback loop in *Medicago*

Zhang *et al*.

This PDF file includes:

Supplementary Figures 1 to 37

Supplementary Tables 1 to 3

Supplementary References

Supplementary Figures

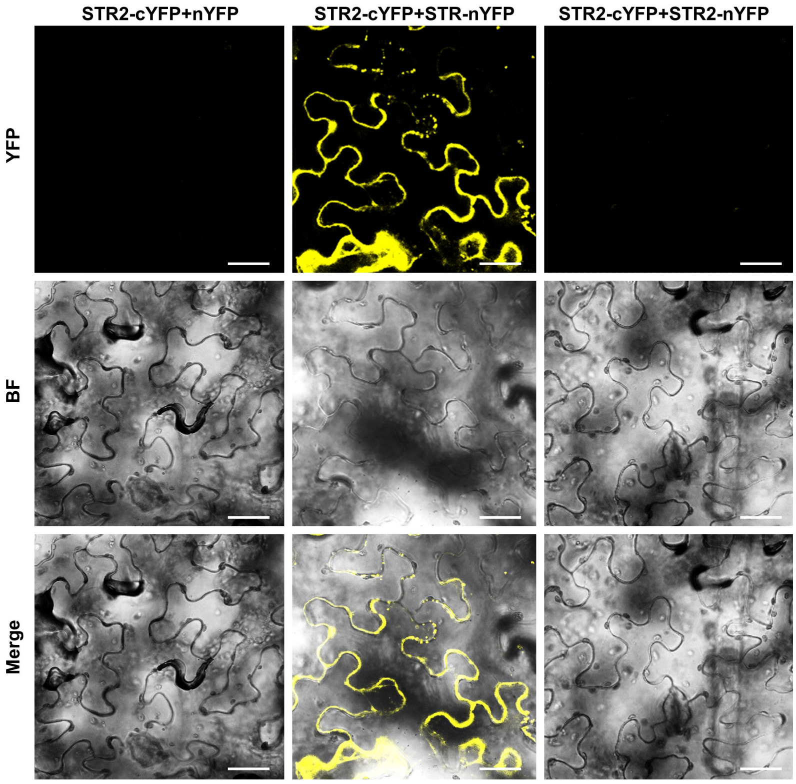

Supplementary Figure 1. BiFC assay showing the interaction between *Medicago* STR2 and STR in *N. benthamiana* leaves. The YFP fluorescence signals of the leaves co-transformed with indicated BiFC combinations were detected. nYFP, N-terminal fragment of YFP; cYFP, C-terminal fragment of YFP; BF: Bright-field; Merge: YFP + BF. Scale bar, 50 μm.

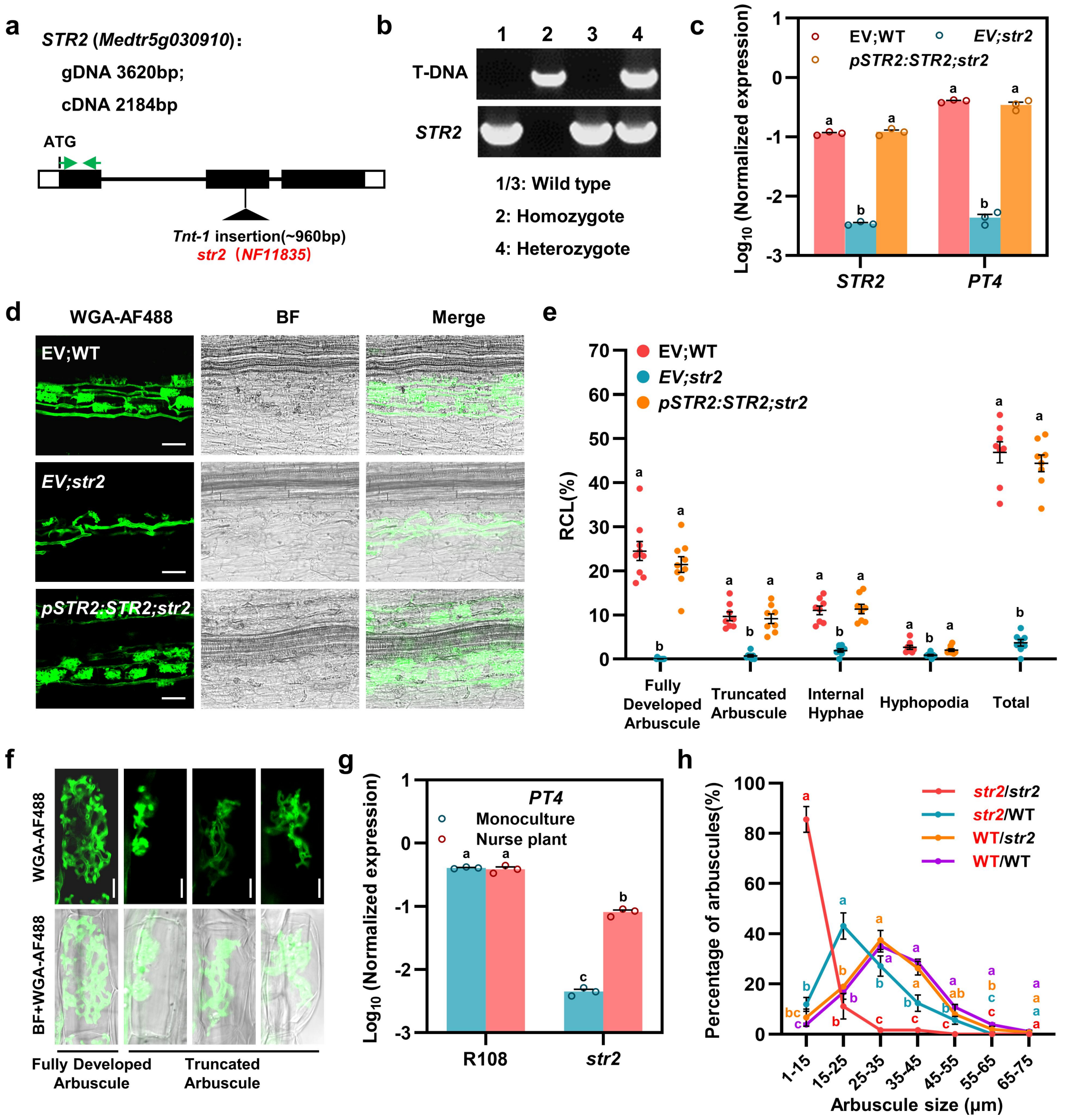

Supplementary Figure 2. ***Medicago STR2*** **is indispensable for arbuscule development during AM symbiosis.** (**a**) Schematic representation of the *str2* mutant allele (*NF11835*). The *Tnt1* insertion site is indicated by a triangle. The black boxes, lines, and white boxes indicate the exon, intron, and the 5′/3′-UTR regions, respectively. Green arrows indicate the primers specifically used for quantifying the *STR2* expression levels. (**b**) Identification of homozygous mutant lines by PCR genotyping. Lanes 1 and 3, Wild type (WT). Lane 2, Homozygous plant. Lane 4, Heterozygous plant. (**c-e**) Relative expression levels of *STR2* and *PT4* (**c**), images of WGA-AF488-stained arbuscules (**d**), and quantification of *R. irregularis* colonisation level (**e**) for indicated genotypes at 6 weeks post-inoculation with *R. irregularis* (wpi). Relative expression was normalised to that of *MtEF-1*. EV, empty vector. Scale bar, 50 μm. (**f**) Representative images of the fully developed arbuscule and truncated arbuscule. The arbuscules were divided into two types according to their relative size ratios to cortical cells as mentioned in the methods section. Scale bar, 10 μm. (**g**) Relative expression levels of *PT4* in WT and *str2* grown as a monoculture or with a WT nurse plant at 6 wpi. Relative expression

was normalised to that of *MtEF-1*. (**h**) Arbuscule size distribution of *str2* and WT plants grown with a *str2* or WT nurse plant at 6 wpi. The tester plant is labelled in red, and the nurse plant is labelled in black. Statistics: Individual data points (**c**, **e**, **g**) and mean ± SE are shown. Different letters indicate significant differences (One-way ANOVA, Duncan's multiple range test, $P < 0.05$). Exact *P* values are provided in Source Data. **c** $n = 3$ technical replicates; **e** $n = 9$ independent plants; **g** $n = 3$ biological replicates; **h** $n = 3$ biological replicates from about 200 arbuscules of each genotype.

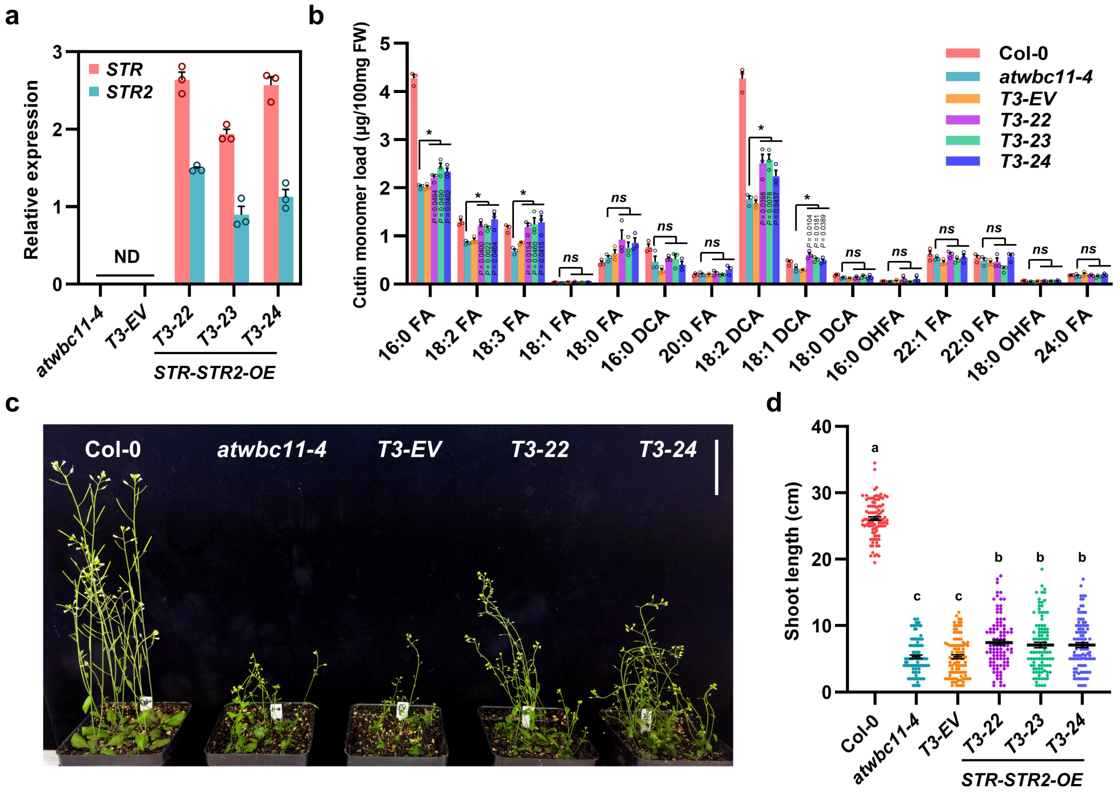

Supplementary Figure 3. Co-overexpression of *Medicago STR* and *STR2* increased cutin monomer accumulation and partially rescued the phenotype of reduced plant height in *atwbc11* plants. (**a**) Relative expression levels of *STR* and *STR2* in leaves of *Arabidopsis atwbc11-4* mutants, empty vector controls (T3-EV), and *STR-STR2* co-overexpressing (T3-22, T3-23, and T3-24) transformed *atwbc11-4* mutants T3 lines. Relative expression was normalised by using *PP2A*. ND, not detectable. The data were obtained from three biological replicates and are presented as mean ± SE. (**b-d**) Cutin analysis of monomer load (**b**), developmental phenotypes (**c**), and shoot length (**d**) for indicated *Arabidopsis* genotypes after 5 weeks of growth in a growth room at 22 ºC with a long-day photoperiod (16-h light/8-h dark cycle). FW, fresh weight. Scale bar, 5 cm. Statistics: Individual data points and mean ± SE are shown. **b** Two-sided Student's *t*-test was used. *$P < 0.05$; *ns*, not significant. $n = 3$ biological replicates. **d** Different letters indicate significant differences (One-way ANOVA, Duncan's multiple range test, $P < 0.05$). Exact P values are provided in Source Data. $n = 100$ independent plants.

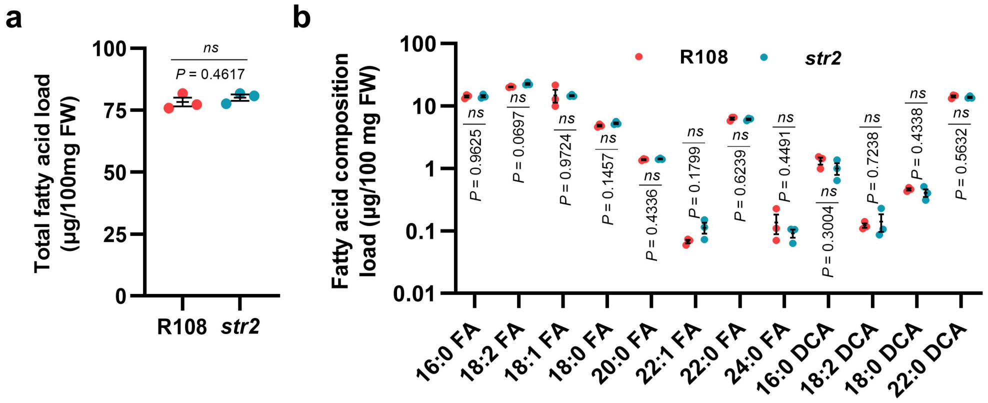

Supplementary Figure 4. Total fatty acid content (a) and fatty acid composition (b) of roots from 4-week-old WT (R108) and *str2* seedlings grown in sand/perlite (1:1) without mycorrhizal fungal infection. FW, fresh weight. Statistics: Individual data points and mean ± SE are shown. *n* = 3 biological replicates from 20 independent plants. Two-sided Student's *t*-test was used. *ns*, not significant. These experiments were repeated three times with similar results.

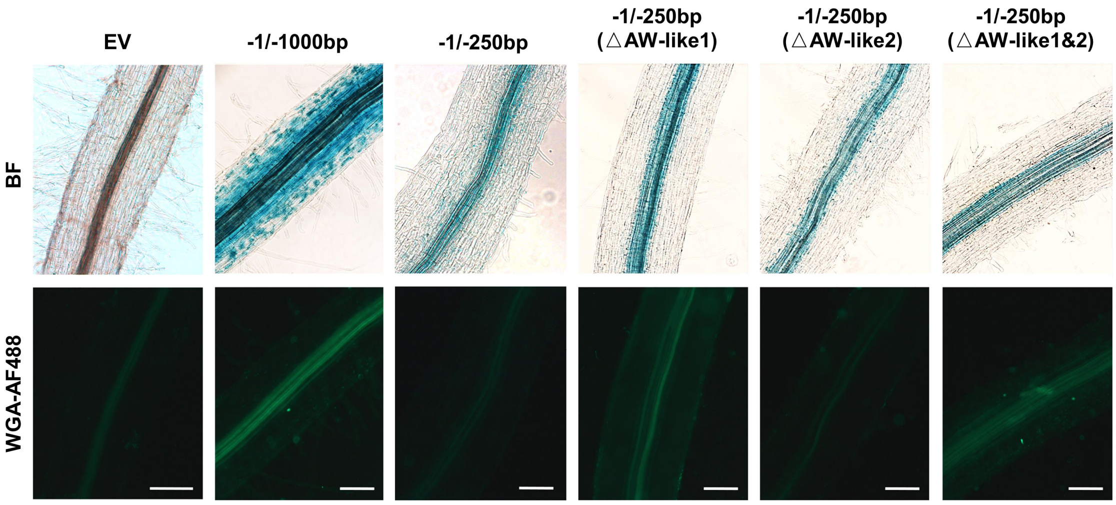

Supplementary Figure 5. Bright-field images (up) and corresponding fluorescence microscopy images (down) of *Medicago* roots without *R. irregularis* colonisation reveal GUS staining corresponding to Fig. 2b. The *GUS* expression in hairy roots was driven by the different lengths (-1/-1000 bp, -1/-250 bp upstream of ATG start codon) or forms (-1/-250 bp with AW-box-like1, AW-box-like2, AW-box-like1&2 deletion upstream of ATG start codon) of the *STR2* promoter. Scale bar, 100 μm. Experiments were repeated 3 times with similar results.

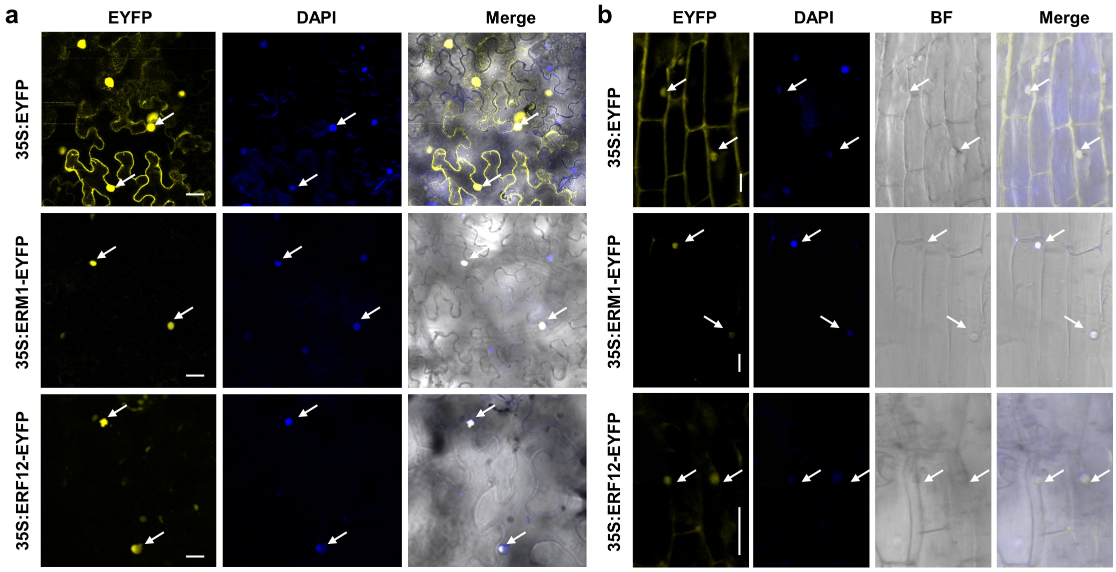

Supplementary Figure 6. ERM1 and ERF12 are specifically localised to the nucleus, both in *N. benthamiana* leaves and in *Medicago* hairy roots. (**a**) ERM1 and ERF12 were localised to the nucleus in *N. benthamiana* leaves. Free YFP and YFP fusions to the ERM1 and ERF12 proteins are shown in yellow. Scale bar, 25 µm. (**b**) Subcellular localisation of the above protein fusions expressing in transgenic *Medicago* hairy roots under the control of the *35S* promoter was examined. Scale bar, 50 µm. White arrows indicate the nucleus sites.

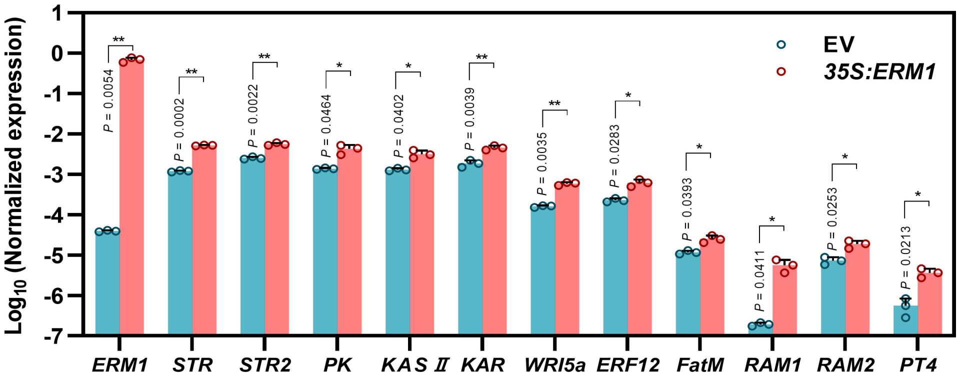

Supplementary Figure 7. ***ERM1*** **overexpression activates the expression of AM symbiosis-related genes.** Relative expression levels of indicated genes were detected in *35S:ERM1* overexpressing and EV *M. truncatula* hairy roots without inoculation with AM fungi. Relative expression was normalised to that of *MtEF-1*. Individual data points and mean ± SD are shown. Two-sided Student's *t*-test was used. *$P < 0.05$; **$P < 0.01$. $n = 3$ technical replicates. These experiments were repeated three times with similar results.

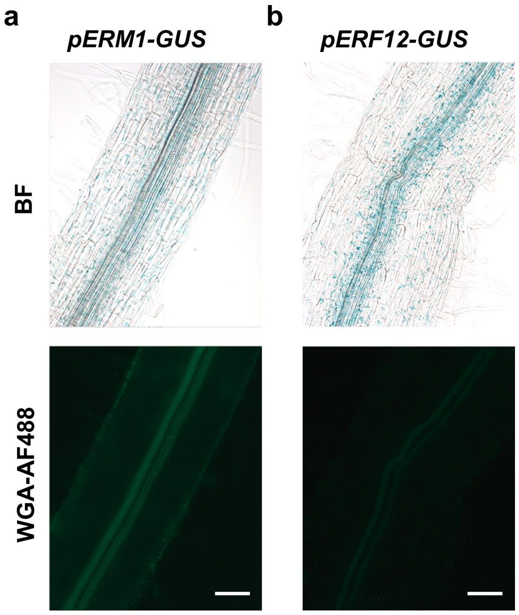

Supplementary Figure 8. Bright-field images (up) and corresponding fluorescence microscopy images (down) of roots without *R. irregularis* colonisation reveal GUS staining corresponding to Figs. 3b and 4b. The *GUS* expression in hairy roots was driven by the 1 kb (upstream of ATG start codon) of the *ERM1* (**a**) and *ERF12* (**b**) promoter. Scale bar, 50 μm. Experiments were repeated 3 times with similar results.

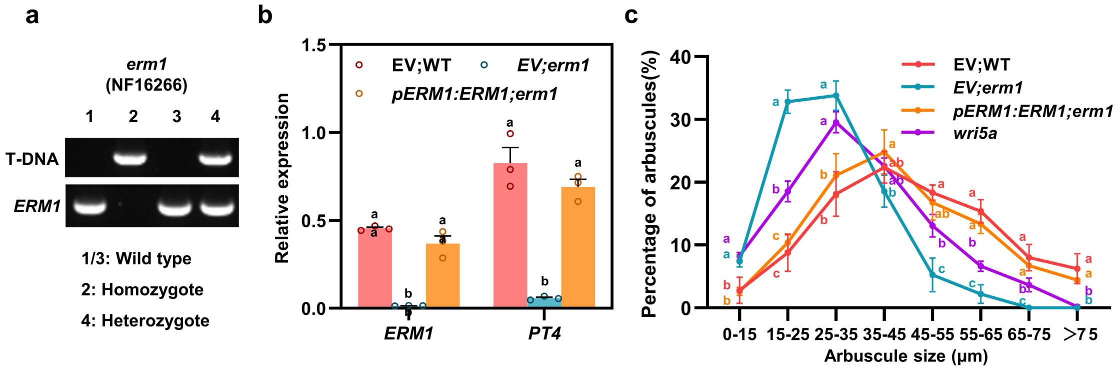

Supplementary Figure 9. ***Medicago ERM1*** **is required for arbuscule development. (a)** Identification of homozygous mutant lines by PCR genotyping. Lanes 1 and 3, Wild type. Lane 2, Homozygous plant. Lane 4, Heterozygous plant. (**b**) Relative expression levels of *ERM1* and *PT4* for indicated genotypes at 6 wpi. Relative expression was normalised by using *MtEF-1*. (**c**) Arbuscule size distribution for indicated genotypes at 6 wpi. Statistics: Individual data points (**b**) and mean ± SE are shown. Different letters indicate significant differences (One-way ANOVA, Duncan's multiple range test, $P < 0.05$). Exact P values are provided in Source Data. **b** $n = 3$ biological replicates. **c** $n = 3$ biological replicates from about 200 arbuscules of each genotype. These experiments were repeated three times with similar results.

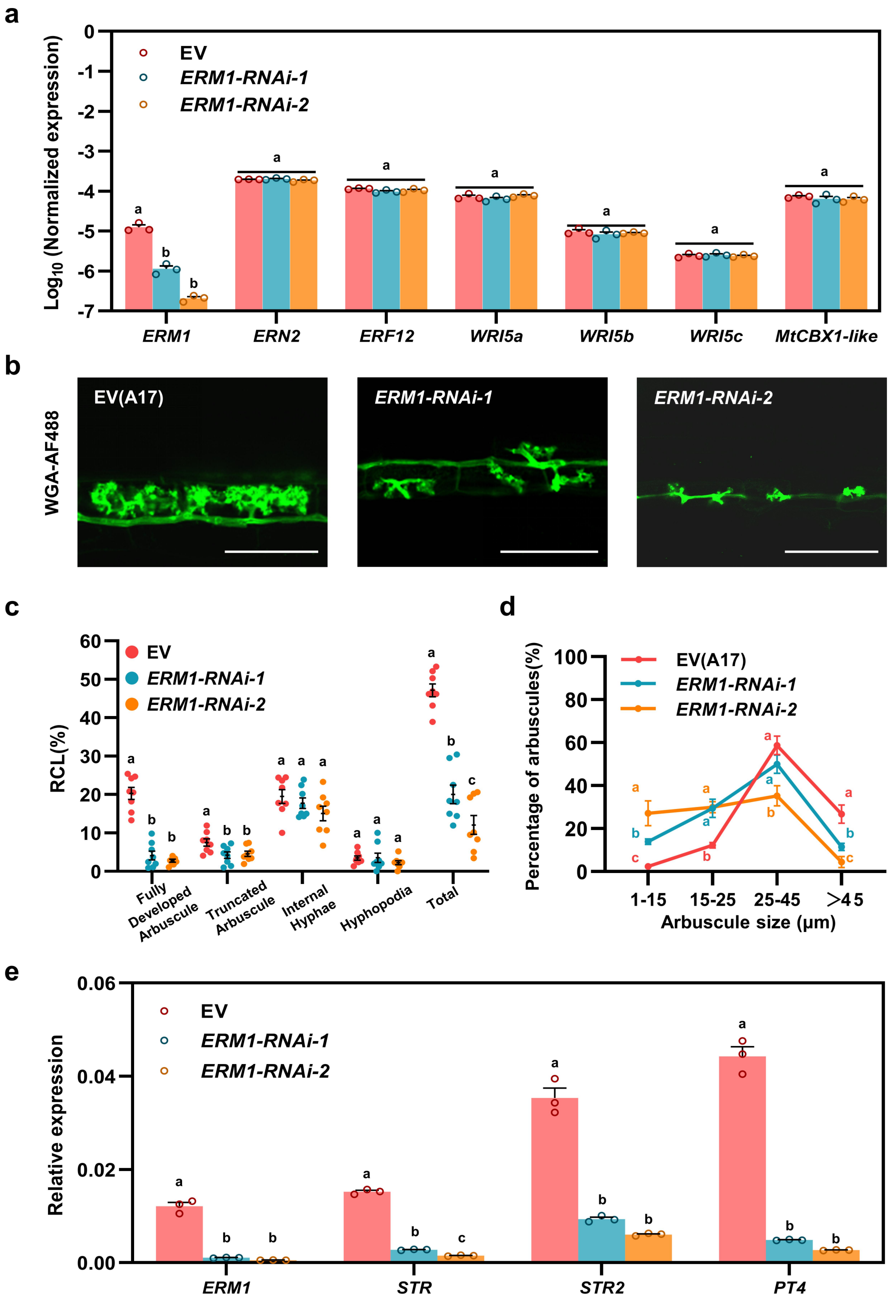

Supplementary Figure 10. Impaired arbuscule development in *ERM1-RNAi* transformed *M. truncatula* hairy roots. (a) Relative expression levels of indicated genes in *ERM1-RNAi-1*, *ERM1-RNAi-2*, and EV (A17 background) *M. truncatula* hairy roots without inoculation with AM fungi. The predicted ortholog of *LjCBX1* in *M. truncatula* is named *MtCBX1-like* (*Medtr2g460730*). Relative expression was normalised by using *MtEF-1*. (**b-e**) Images of WGA-AF488-stained arbuscules (**b**), quantification of *R. irregularis* colonisation level (**c**), arbuscule size distribution (**d**), and relative expressions of *ERM1*/*STR*/*STR2*/*PT4* (**e**) for indicated genotypes upon 6 wpi. Scale bar, 50 μm. Relative expression was normalised by using *MtEF-1*. Statistics: Individual data points (**a**, **c**, **e**) and mean ± SE are shown. Different letters indicate significant differences (One-way ANOVA, Duncan's multiple range test, $P < 0.05$). Exact *P* values are provided in Source Data. **a** $n = 3$ technical replicates;

c n = 8 independent plants; **d** n = 3 biological replicates from about 200 arbuscules of each genotype. **e** n = 3 technical replicates. These experiments were repeated three times with similar results.

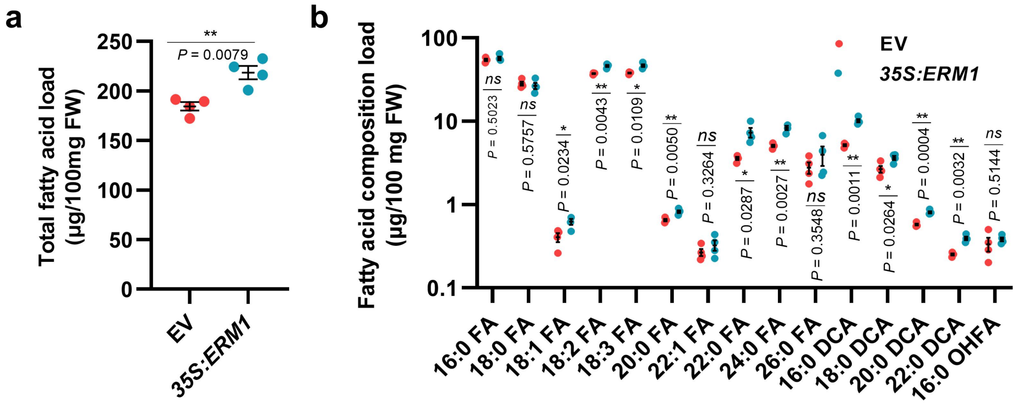

Supplementary Figure 11. *ERM1* contributes to fatty acid accumulation in *Medicago* roots. (**a**–**b**) Total fatty acid content (**a**) and fatty acid composition (**b**) in *35S:ERM1*-overexpression and EV (A17) *M. truncatula* hairy roots without mycorrhizal fungal infection. FW, fresh weight. Statistics: Individual data points and mean ± SE are shown. n = 4 biological replicates from 15 independent plants. Two-sided Student's t-test was used. *$P < 0.05$; **$P < 0.01$; *ns*, not significant.

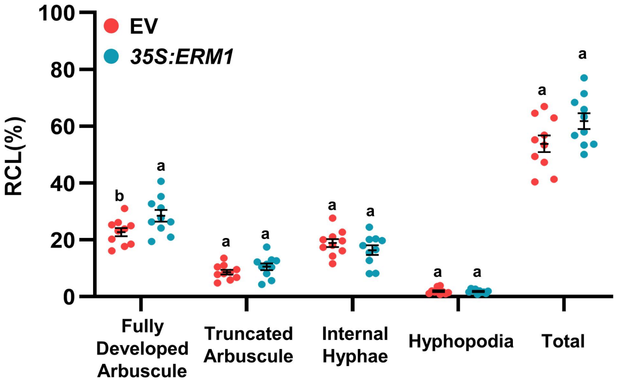

Supplementary Figure 12. Quantification of AM colonisation in *35S:ERM1* and EV (A17) *M. truncatula* hairy roots at 5 wpi. Statistics: Individual data points and mean ± SE are shown. Different letters indicate significant differences (One-way ANOVA, Duncan's multiple range test, $P < 0.05$). Exact P values are provided in Source Data. $n = 10$ independent plants. This experiment was repeated three times with similar results.

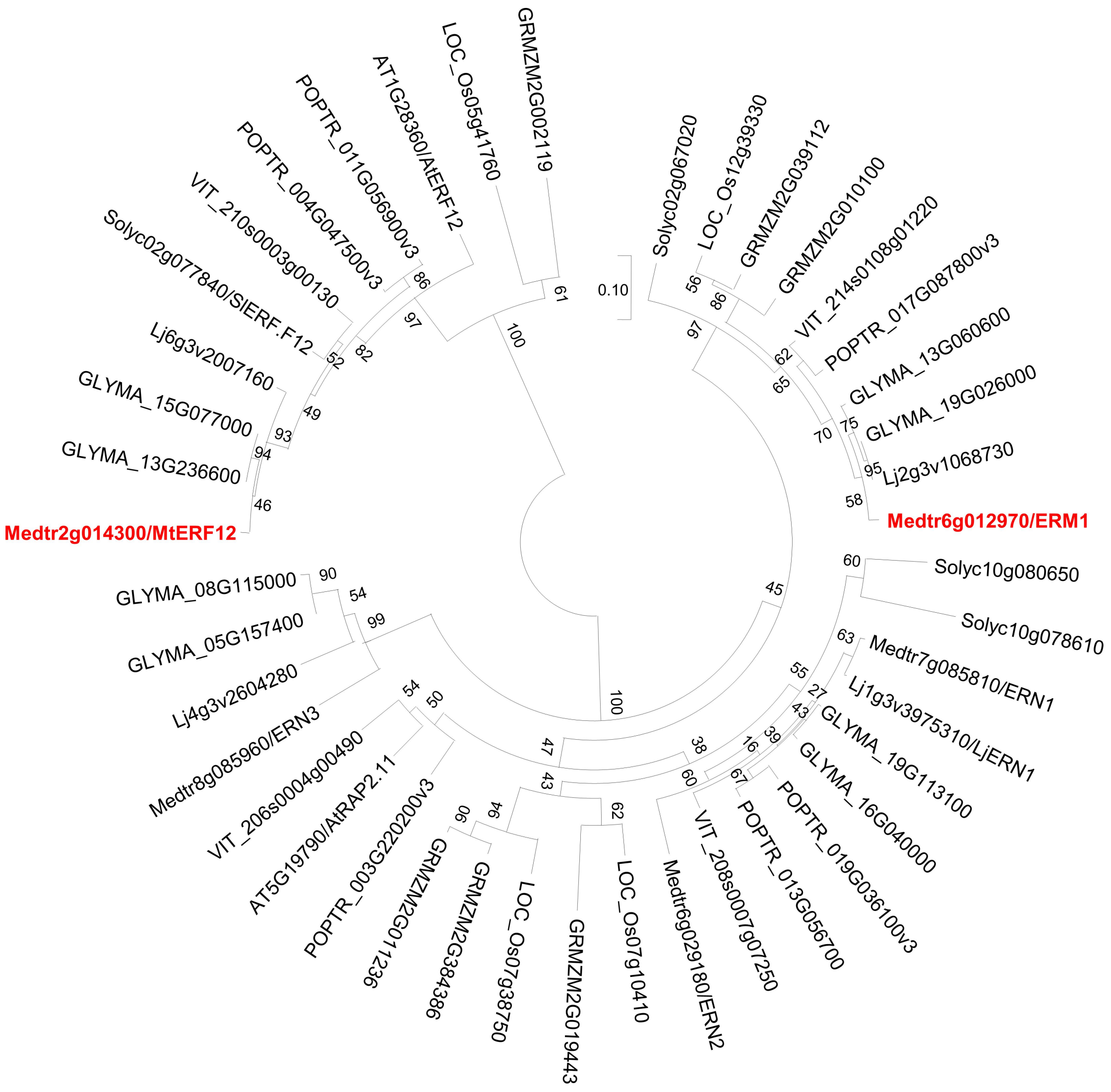

Supplementary Figure 13. Phylogenetic tree of ERF subfamily transcription factors from *A. thaliana* (AT), *M. truncatula* (Medtr), *Glycine max* (GLYMA), *Lotus japonicus* (Lj), *Oryza sativa* (Os), *Populus trichocarpa* (POPTR), *Zea mays* (GRMZM), *Vitis vinifera* (VIT), and *Solanum lycopersicum* (Solyc), with a focus on ERM1 and ERF12 and their orthologs. The tree was constructed by MEGA 11 software after 1000 bootstrap calculations using the maximum likelihood method. The bootstrap values for the branches are shown.

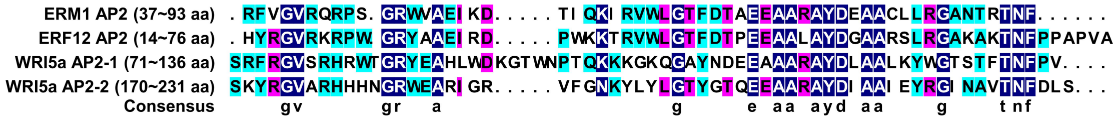

Supplementary Figure 14. Consensus sequence alignment of the AP2 domains from ERM1, ERF12, and WRI5a proteins. Dark blue boxes indicate conserved amino acids between four AP2 domains. This was constructed by DNAMAN 7 using default parameters.

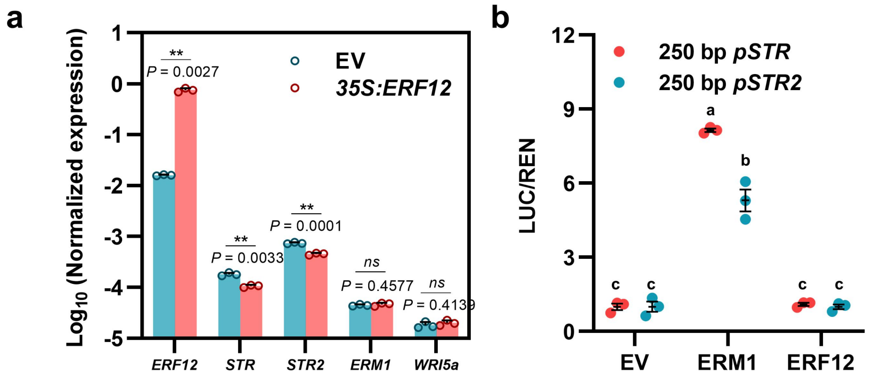

Supplementary Figure 15. ERF12 cannot transactivate the 250-bp *STR* and *STR2* promoters. (a) Relative expression levels of indicated genes in *M. truncatula* hairy roots (A17) transformed via *35S:ERF12* and empty vector (EV). Relative expression was normalised to that of *MtEF-1*. **(b)** *In vivo* transcriptional activation assay using a dual-luciferase system showing the activation of the 250-bp *STR/STR2* promoters by ERM1 and ERF12, which acted as effectors. Statistics: Individual data points and mean ± SE are shown. **a** Two-sided Student's *t*-test was used. $^{**}P < 0.01$; *ns*, not significant. $n = 3$ technical replicates. **b** Different letters indicate significant differences (One-way ANOVA, Duncan's multiple range test, $P < 0.05$). Exact P values are provided in Source Data. $n = 3$ biological replicates.

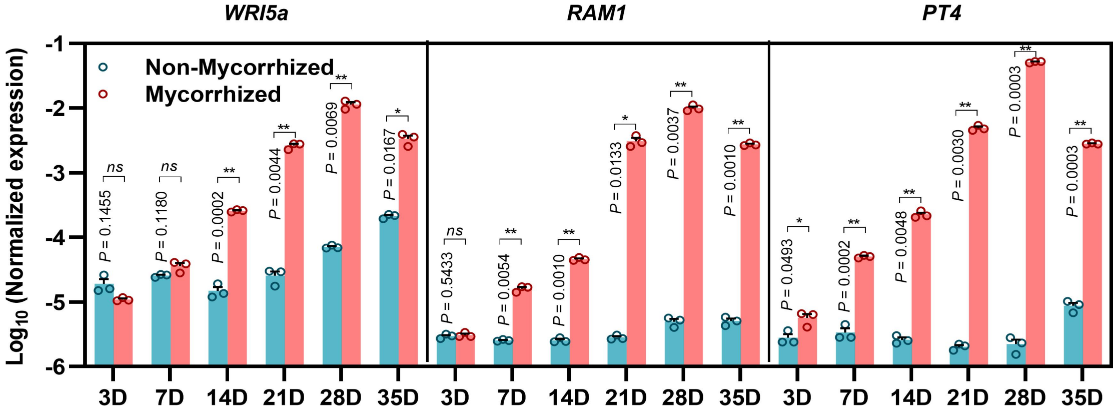

Supplementary Figure 16. AM symbiosis marker genes were upregulated during mycorrhizal fungal infection. Relative expression levels of *WRI5a*, *RAM1*, and *PT4* in *Medicago* R108 roots at 3, 7, 14, 21, 28, and 35 days (D) post-inoculation with *R. irregularis*. Relative expression was normalised by using *MtEF-1*. Statistics: Individual data points and mean ± SE are shown. $n = 3$ technical replicates. Two-sided Student's *t*-test was used. *$P < 0.05$; **$P < 0.01$; *ns*, not significant.

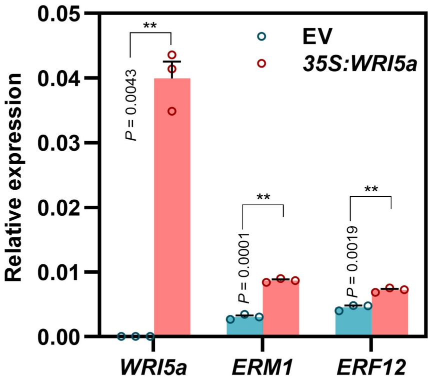

Supplementary Figure 17. WRI5a regulates *ERM1* and *ERF12* at the transcriptional level. Relative expression levels of *WRI5a*, *ERM1*, and *ERF12* in *35S:WRI5a* and EV control *M. truncatula* hairy roots (A17) without inoculation with AM fungi. Relative expression was normalised to that of *MtEF-1*. Statistics: Individual data points and mean ± SE are shown. n = 3 technical replicates. Two-sided Student's t-test was used. $^{**}P < 0.01$. These experiments were repeated three times with similar results.

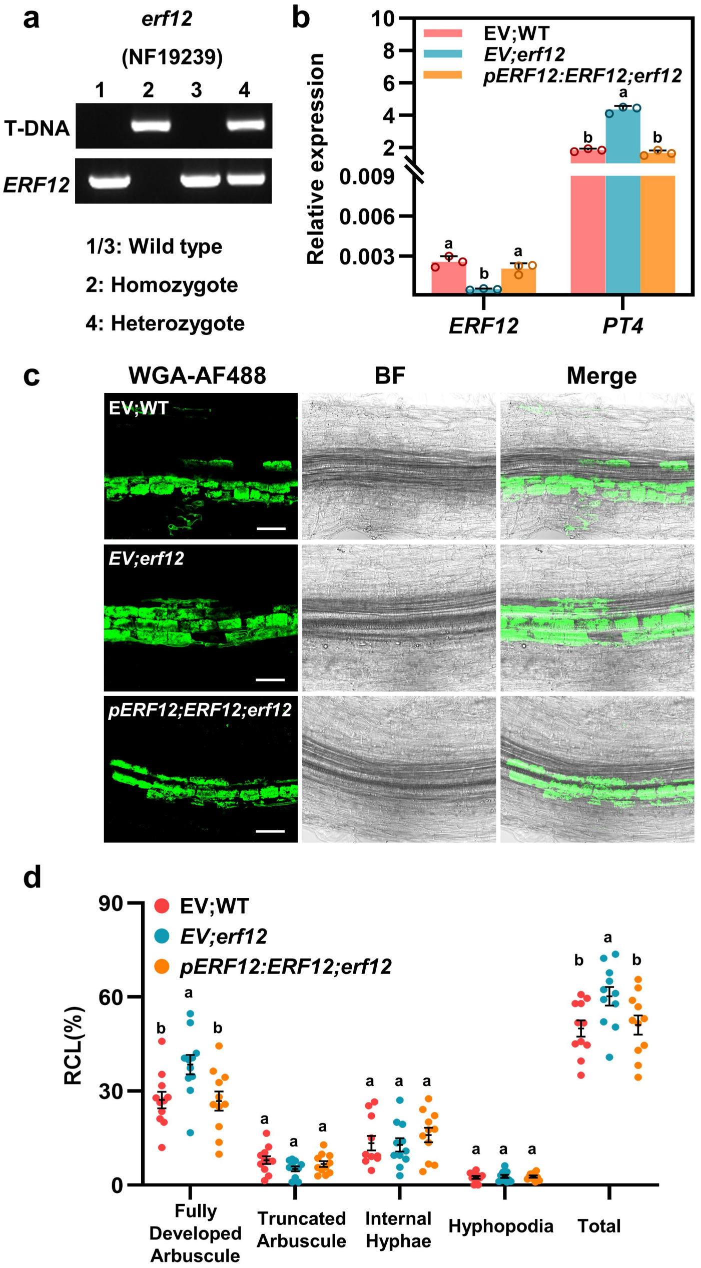

Supplementary Figure 18. ERF12 negatively regulates arbuscule development. (**a**) Identification of homozygous mutant lines by PCR genotyping. Lanes 1 and 3, Wild type. Lane 2, Homozygous plant. Lane 4, Heterozygous plant. (**b**) Relative expression levels of *ERF12* and *PT4* for indicated genotypes at 6 wpi. Relative expression was normalised by using *MtEF-1*. (**c-d**) Images of WGA-AF488-stained arbuscules (**c**) and quantification of *R. irregularis* colonisation level (**d**) for indicated genotypes at 6 wpi. Scale bar, 50 μm. Statistics: Individual data points and mean ± SE are shown. Different letters indicate significant differences (One-way ANOVA, Duncan's multiple range test, $P < 0.05$). Exact *P* values are provided in Source Data. **b** $n = 3$ biological replicates. **d** $n = 10-12$ independent plants. These experiments were repeated three times with similar results.

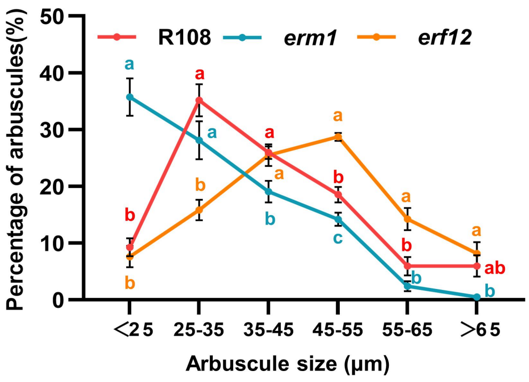

Supplementary Figure 19. Arbuscule size distribution for R108, *erm1*, and *erf12* at 5 wpi corresponding to Fig. 4g. Statistics: Values are means ± SE. Different letters indicate significant differences (One-way ANOVA, Duncan's multiple range test, $P < 0.05$). Exact P values are provided in Source Data. $n = 3$ biological replicates from about 200 arbuscules of each genotype.

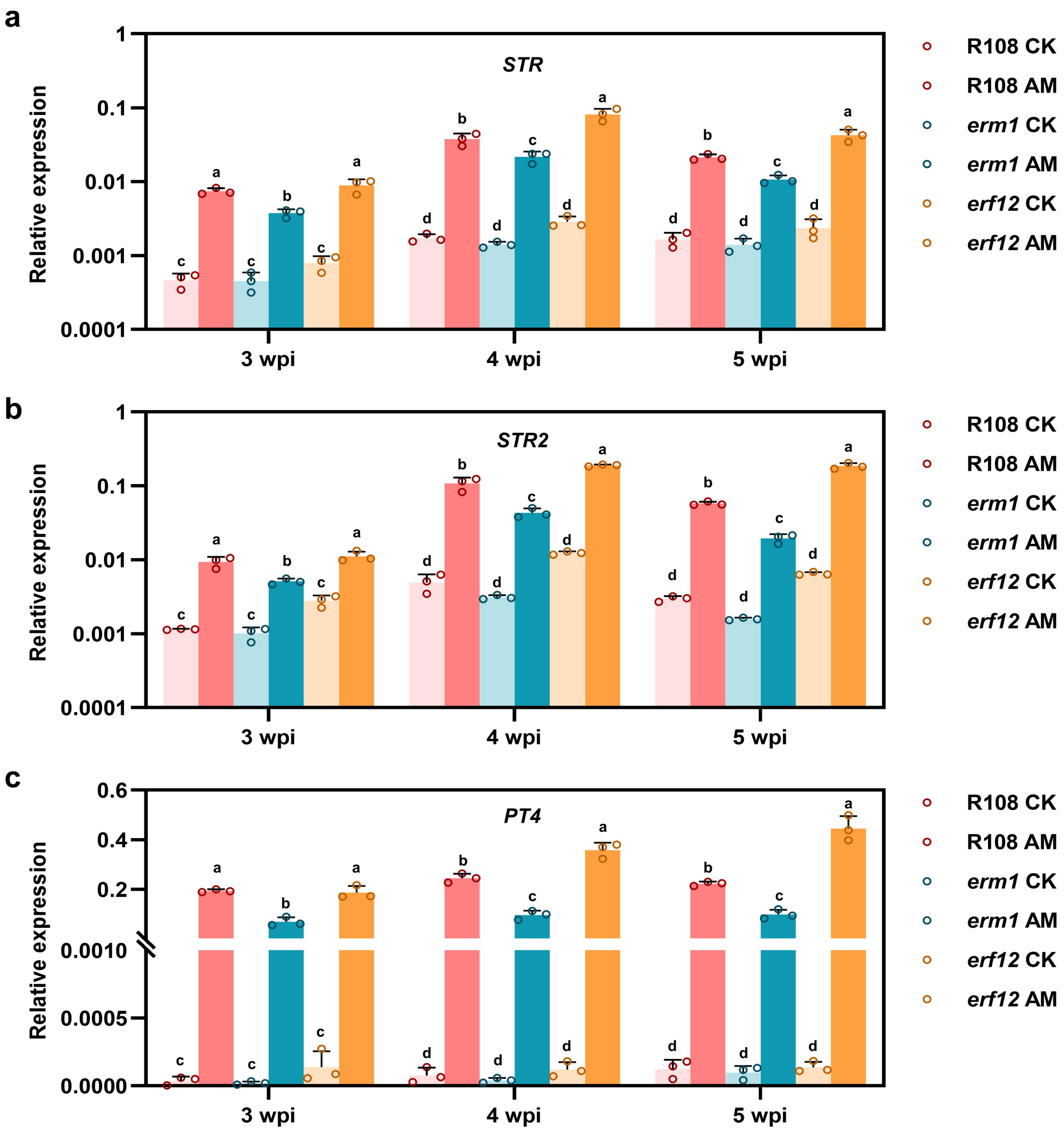

Supplementary Figure 20. Relative expression levels of *STR* (a), *STR2* (b), and *PT4* (c) in R108, *erm1*, and *erf12* roots without (CK) or with (AM) inoculation with AM fungi corresponding to Fig. 4g. Relative expression was normalised by using *MtEF-1*. Statistics: Individual data points and mean ± SD are shown. Different letters indicate significant differences (One-way ANOVA, Duncan's multiple range test, $P < 0.05$). Exact P values are provided in Source Data. $n = 3$ technical replicates. These experiments were repeated three times with similar results.

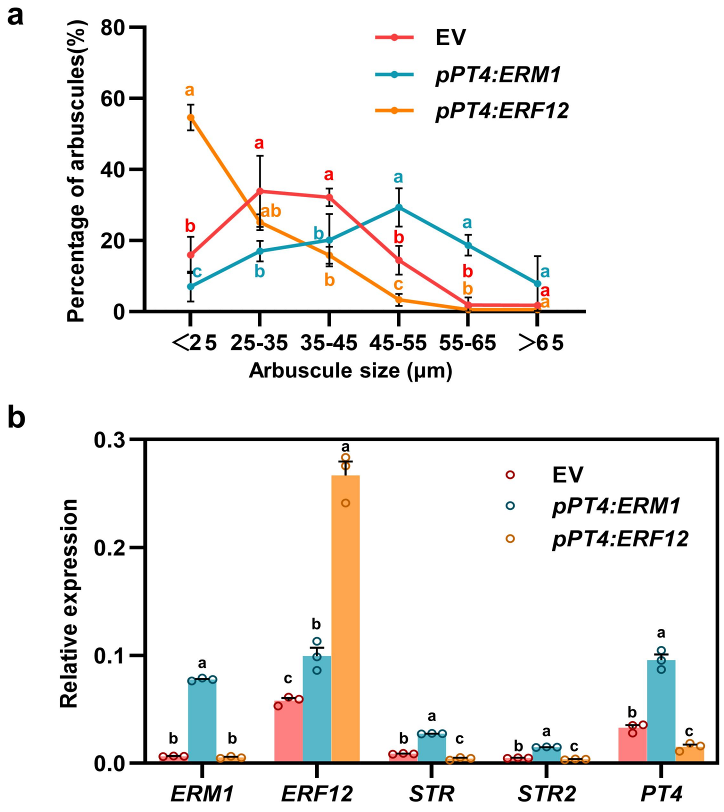

Supplementary Figure 21. Arbuscule size distribution (a) and relative expression levels of indicated genes (b) for p*PT4:ERF12*, p*PT4:ERM1*, and EV transformed *M. truncatula* hairy roots (A17) at 5 wpi corresponding to Fig. 4j. Relative expression was normalised by using *MtEF-1*. Statistics: Individual data points (**b**) and mean ± SE are shown. Different letters indicate significant differences (One-way ANOVA, Duncan's multiple range test, $P < 0.05$). Exact P values are provided in Source Data. **a** $n = 3$ biological replicates from about 200 arbuscules of each genotype. **b** $n = 3$ technical replicates. These experiments were repeated twice with similar results.

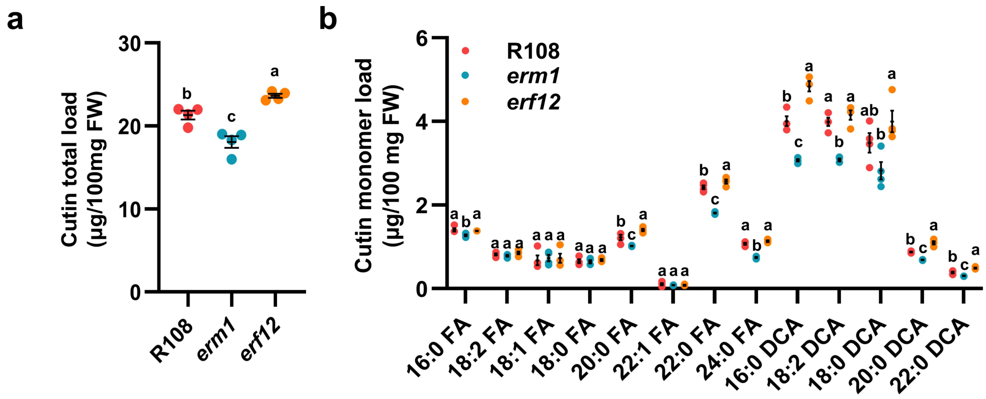

Supplementary Figure 22. Total cutin load (a) and cutin monomer load (b) of roots from 6-week-old WT (R108), *erm1*, and *erf12* seedlings grown in sand/perlite (1:1) without AM fungal infection. FW, fresh weight. Statistics: Individual data points and mean ± SE are shown. Different letters indicate significant differences (One-way ANOVA, Duncan's multiple range test, $P < 0.05$); Exact P values are provided in Source Data. $n = 4$ biological replicates from 15 plants.

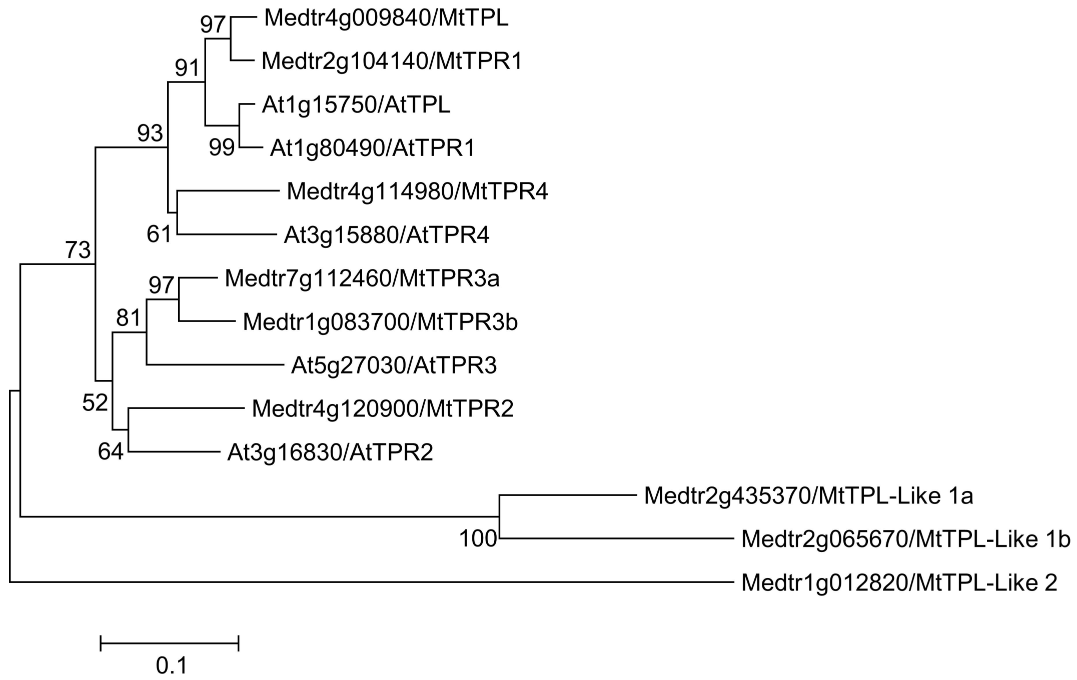

Supplementary Figure 23. A phylogenetic tree of the *TPL/TPR* genes was constructed using homologs from *A. thaliana* (At) and *M. truncatula* (Medtr). The tree was constructed by MEGA 4 software after 1000 bootstrap calculations using the maximum likelihood method. The bootstrap values for the branches are shown.

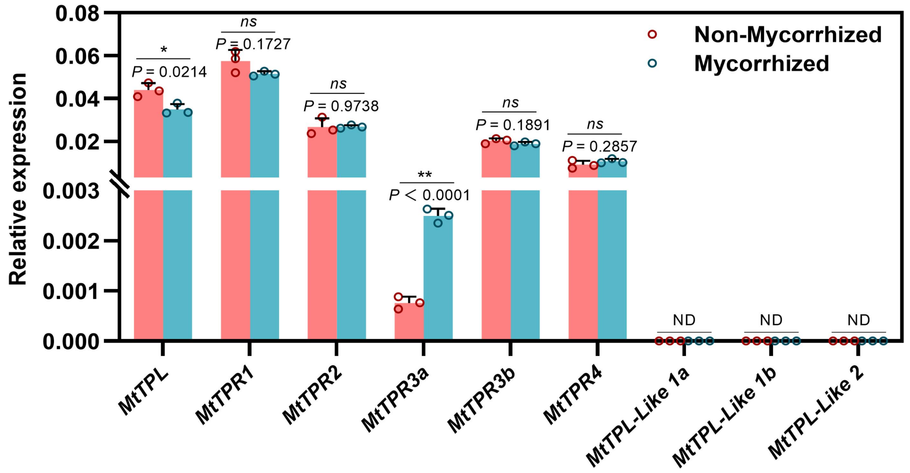

Supplementary Figure 24. Relative expression levels of *TPL*/*TPR*-like genes in non-mycorrhized and mycorrhized *Medicago* R108 roots at 4 wpi. Relative expression was normalised to that of *MtEF-1*. Statistics: Individual data points and mean ± SD are shown. $n = 3$ technical replicates. Two-sided Student's *t*-test was used. $^*P < 0.05$, $^{**}P < 0.01$; *ns*, not significant. These experiments were repeated three times with similar results.

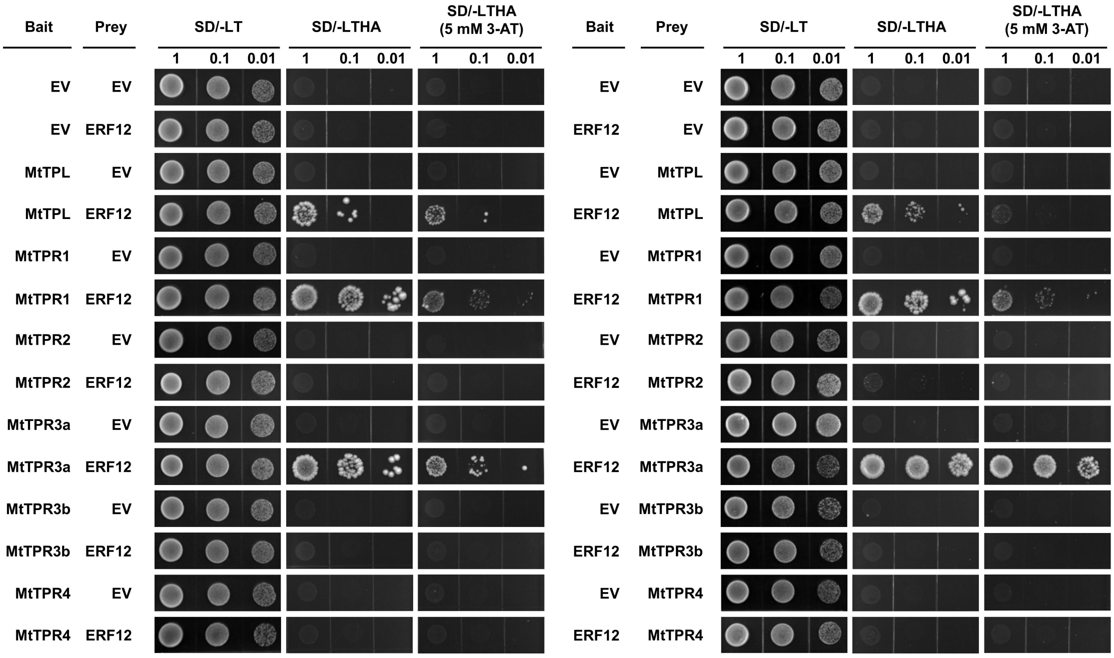

Supplementary Figure 25. ***Medicago*** **ERF12 interacts with MtTPL, MtTPR1, and MtTPR3a in a Y2H assay.** Bait, a protein with binding domain; Prey, a protein with activation domain; EV, empty vector; The numbers on the top of each photograph indicate the different concentrations of yeast cells. SD/-LT, synthetic dropout (SD) media lacking leucine and tryptophan; SD/-LTHA, SD media lacking leucine, tryptophan, histidine, and adenine; 3-AT, 3-amino-1,2,4-triazole. These experiments were repeated three times with similar results.

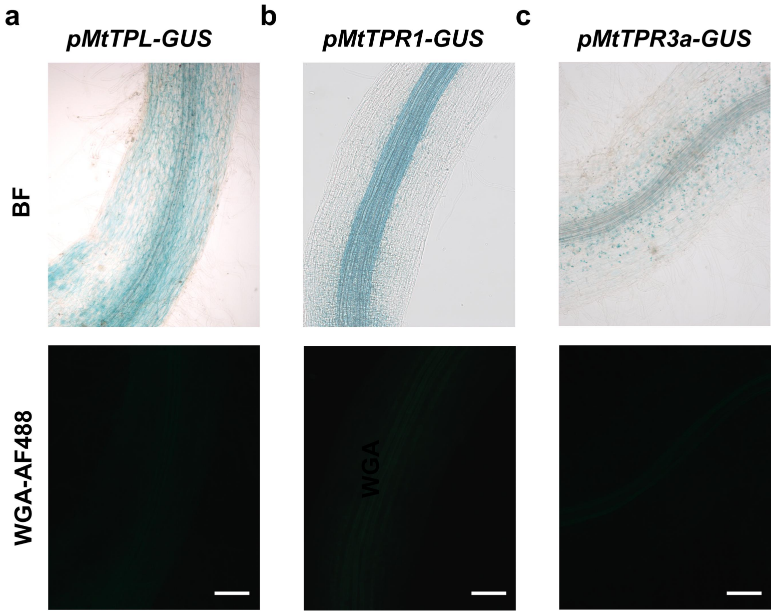

Supplementary Figure 26. Bright-field images (up) and corresponding fluorescence microscopy images (down) of roots without *R. irregularis* colonisation reveal GUS staining corresponding to Fig. 5d. The hairy roots were driven by the 1, 2.1, and 1.6 kb promoter (upstream of ATG start codon) of the *MtTPL* (**a**), *MtTPR1* (**b**), and *MtTPR3a* (**c**), respectively. Scale bar, 50 µm. Experiments were repeated twice with similar results.

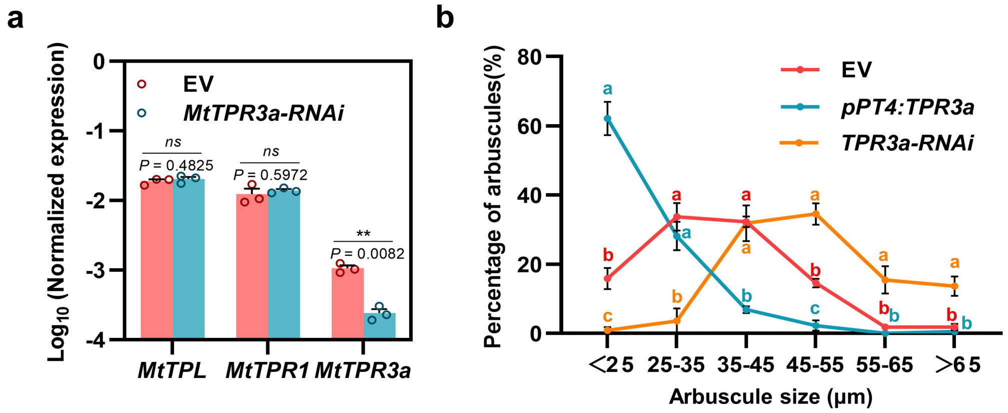

Supplementary Figure 27. Relative expression levels of *MtTPL, MtTPR1, and MtTPR3a* in non-mycorrhized *M. truncatula* hairy roots (A17) transformed via EV and *MtTPR3a-RNAi* (a) and arbuscule size distribution for indicated genotypes (b) at 5 wpi corresponding to Fig. 5e. Relative expression was normalised by using *MtEF-1*. Statistics: Individual data points (**a**) and mean ± SE are shown. **a** $n = 3$ technical replicates. Two-sided Student's *t*-test was used. $^{**}P < 0.01$; *ns*, not significant. **b** $n = 3$ biological replicates from about 200 arbuscules of each genotype. Different letters indicate significant differences (One-way ANOVA, Duncan's multiple range test, $P < 0.05$). Exact P values are provided in Source Data. These experiments were repeated twice with similar results.

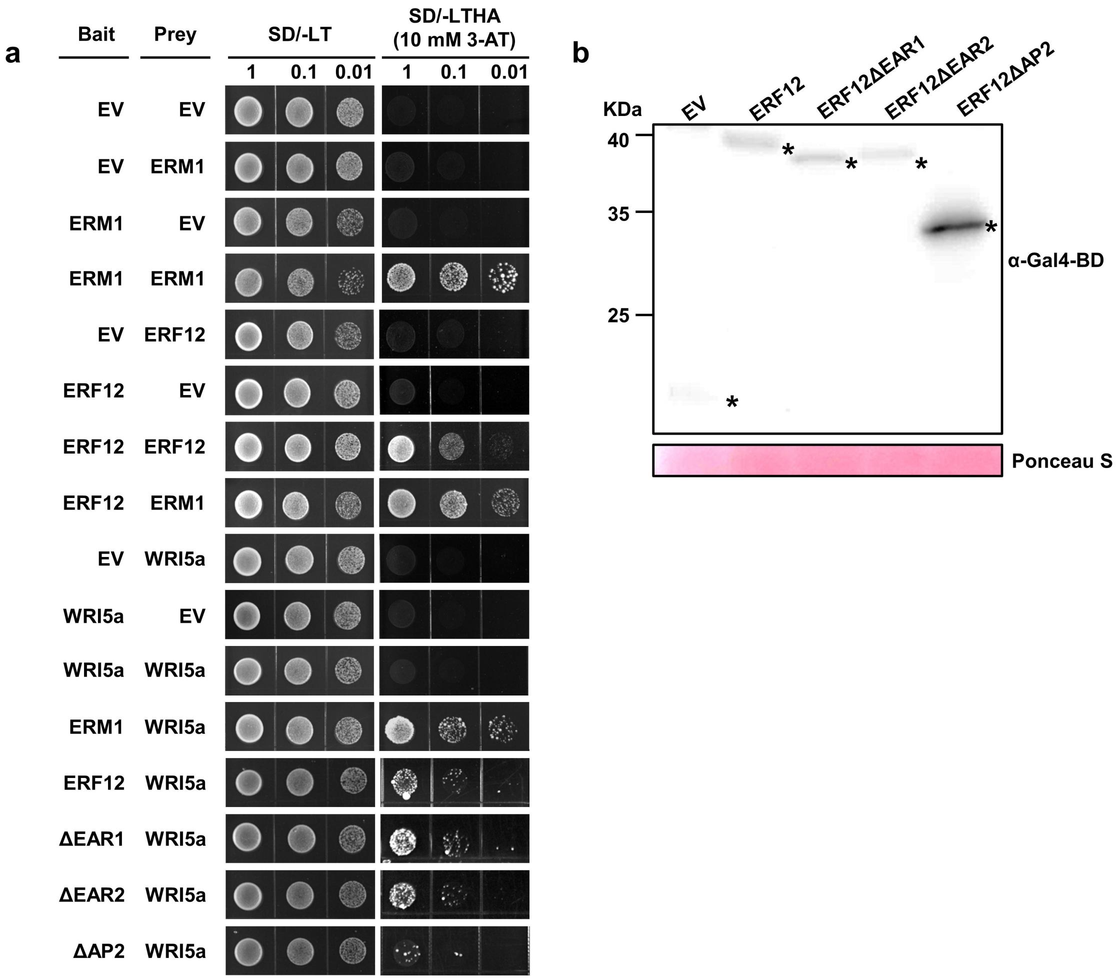

Supplementary Figure 28. ERM1, ERF12, and WRI5a proteins could physically interact with one another and the AP2 domain of ERF12 is required for interaction with WRI5a in a yeast two-hybrid assay. (a) Protein-protein interactions in a yeast two-hybrid assay. Bait, a protein with binding domain; Prey, a protein with activation domain; EV, empty vector; The numbers on the top of each photograph indicate the different concentrations of yeast cells. Full-length (ERF12) and domain-deleted ERF12 protein fragments (ΔEAR1, ΔEAR2, and ΔAP2) were used as bait with WRI5a used as a prey. SD/-LT, synthetic dropout (SD) media lacking leucine and tryptophan; SD/-LTHA, SD media lacking leucine, tryptophan, histidine, and adenine; 3-AT, 3-amino-1,2,4-triazole. (**b**) Immunodetection of protein expression in yeasts showed that ERF12ΔAP2 still accumulated in yeast cells. pGBKT7 (EV), pGBKT7-ERF12, pGBKT7-ERF12ΔEAR1, pGBKT7-ERF12ΔEAR2, and pGBKT7-ERF12ΔAP2 detected using anti-GAL4-BD antibody. The asterisks indicate the target proteins. Ponceau S staining served as loading control. These experiments were repeated three times with similar results.

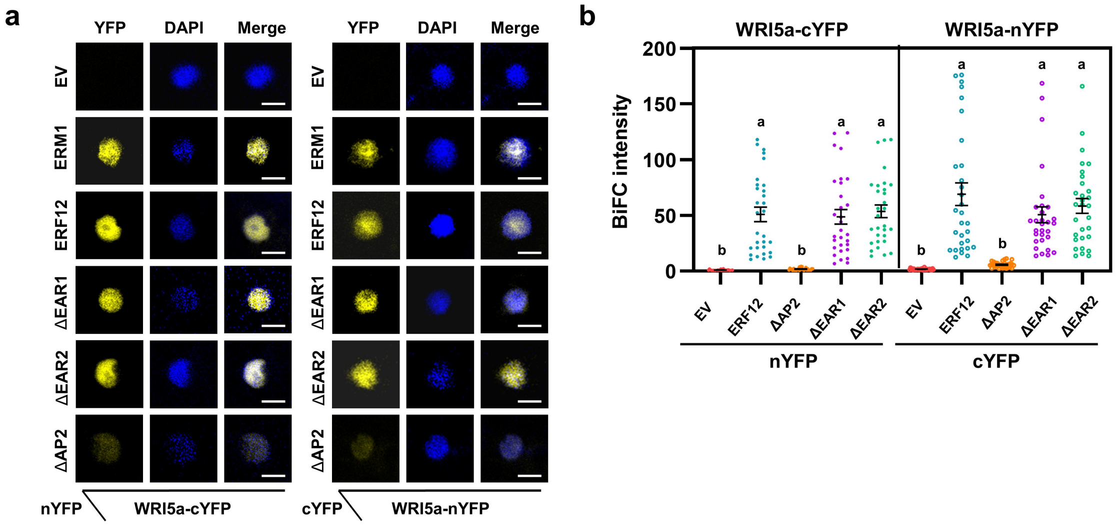

Supplementary Figure 29. BiFC assay showing the protein-protein interactions in *N. benthamiana* leaves. (**a**) YFP fluorescence signals were detected in leaves co-transformed with the indicated BiFC combinations. ΔEAR1, ΔEAR2, and ΔAP2 indicate ERF12 protein fragments in which the corresponding domain has been deleted. nYFP, N-terminal fragment of YFP; cYFP, C-terminal fragment of YFP; DAPI, 4′,6-diamidino-2-phenylindole for nuclear staining; Merge, YFP + DAPI. Scale bar, 5 µm. (**b**) Quantification of YFP signal intensity (Leica Application Suite X 3.3.0 software) from BiFC combinations of WRI5a and ERF12 with various deletions of the indicated domains corresponding to (**a**). Statistics: Individual data points and mean ± SE are shown. Different letters indicate significant differences (One-way ANOVA, Duncan's multiple range test, $P < 0.05$). Exact P values are provided in Source Data. $n = 30$ cells for each BiFC combination.

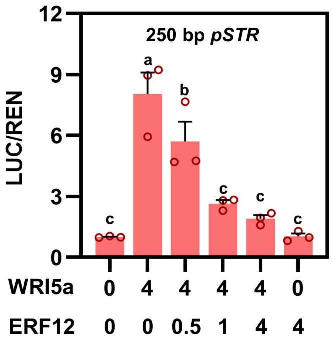

Supplementary Figure 30. ***In vivo*** **transcriptional activation of the 250-bp** ***STR*** **promoter by WRI5a and ERF12 effector mixture assessed with a dual-luciferase system.** Transgenic *Agrobacteria* expressing WRI5a and ERF12 were mixed at different concentrations for infiltration. Statistics: Individual data points and mean ± SE are shown. Different letters indicate significant differences (One-way ANOVA, Duncan's multiple range test, $P < 0.05$). Exact P values are provided in Source Data. $n = 3$ biological replicates. These experiments were repeated three times with similar results.

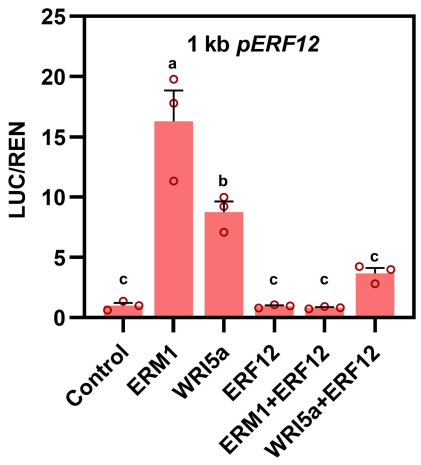

Supplementary Figure 31. ***In vivo*** **transcriptional activation of the** ***ERF12*** **promoter by ERM1/WRI5a/ERF12 alone and their different combinations assessed using a dual-luciferase system.** Statistics: Individual data points and mean ± SE are shown. Different letters indicate significant differences (One-way ANOVA, Duncan's multiple range test, $P < 0.05$). Exact P values are provided in Source Data. $n = 3$ biological replicates. These experiments were repeated three times with similar results.

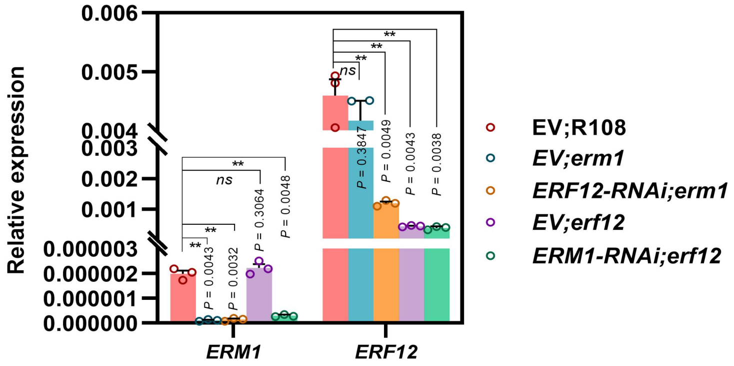

Supplementary Figure 32. Relative expression levels of *ERM1* and *ERF12* in *M. truncatula* hairy roots (A17) without inoculation with AM fungi corresponding to Fig. 6e. Relative expression was normalised by using *MtEF-1*. Statistics: Individual data points and mean ± SE are shown. $n = 3$ technical replicates. Two-sided Student's *t*-test was used. $^{**}P < 0.01$; *ns*, not significant. These experiments were repeated three times with similar results.

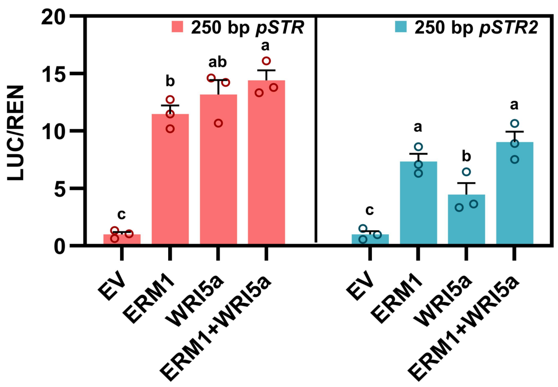

Supplementary Figure 33. *In vivo* transcriptional activation of the 250-bp *STR*/*STR2* promoter by ERM1 or WRI5a alone and their combinations assessed using a dual-luciferase system. Statistics: Individual data points and mean ± SE are shown. Different letters indicate significant differences (One-way ANOVA, Duncan's multiple range test, $P < 0.05$). Exact P values are provided in Source Data. $n = 3$ biological replicates. These experiments were repeated three times with similar results.

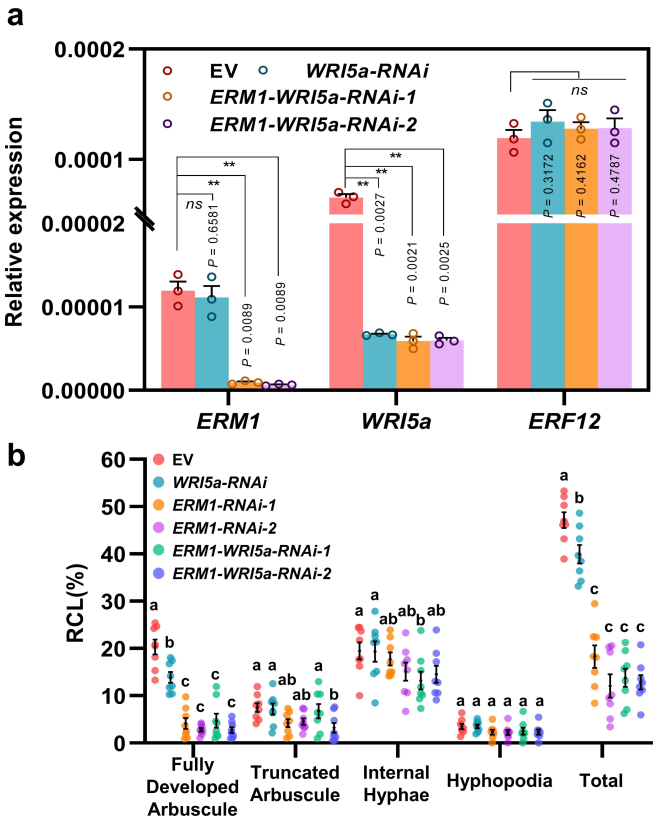

Supplementary Figure 34. *ERM1-WRI5a*-double RNAi plants showed a similar arbuscule development level compared to that of *ERM1*-single RNAi plants. (**a**) Relative expression levels of *ERM1*, *WRI5a*, and *ERF12* in *WRI5a*-RNAi, *ERM1-WRI5a*-RNAi-1, *ERM1-WRI5a*-RNAi-2, and corresponding EV transformed *M. truncatula* hairy roots (A17) without inoculation with AM fungi. Relative expression was normalised by using *MtEF-1*. (**b**) Quantification of AM colonisation level in *M. truncatula* hairy roots (A17) expressing RNAi targeting *WRI5a* (*WRI5a*-RNAi), *ERM1* (*ERM1*-RNAi-1 and *ERM1*-RNAi-2), both *ERM1* and *WRI5a* (*ERM1-WRI5a*-RNAi-1 and *ERM1-WRI5a*-RNAi-2), and the EV at 5 wpi. Statistics: Individual data points and mean ± SE are shown. **a** Two-sided Student's *t*-test was used. $^{**}P < 0.01$; *ns*, not significant. $n = 3$ technical replicates. **b** Different letters indicate significant differences (One-way ANOVA, Duncan's multiple range test, $P < 0.05$). Exact P values are provided in Source Data. $n = 8$ independent plants. These experiments were repeated three times with similar results.

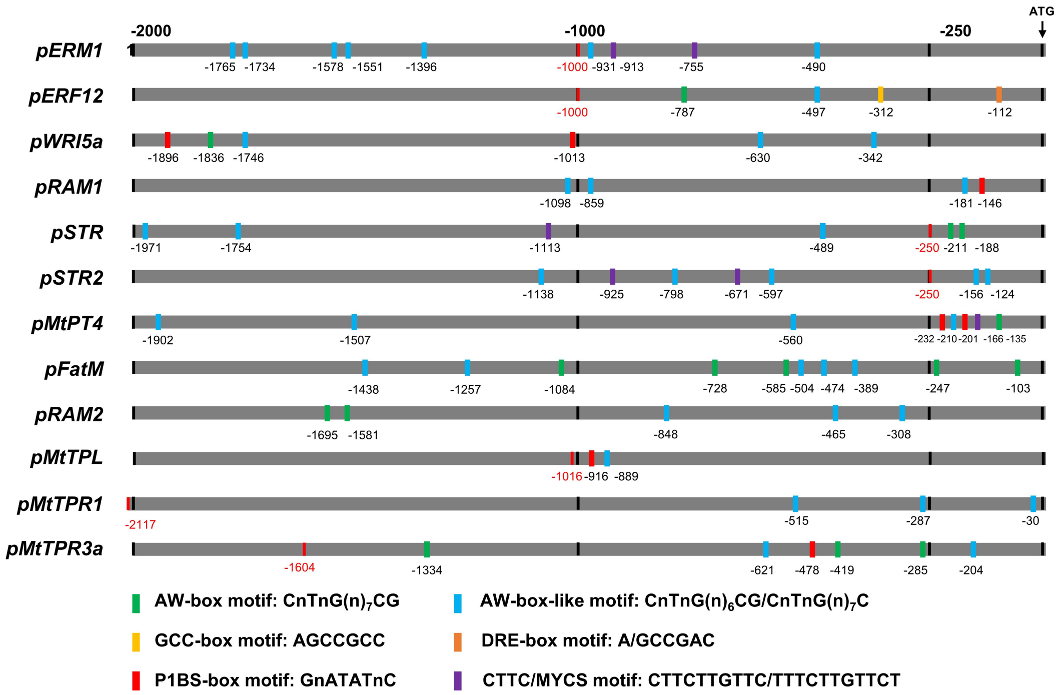

Supplementary Figure 35. Schematic representation of the 2-kb promoter fragments of nutrient exchange-related genes during AM symbiosis and *MtTPL*, *MtTPR1*, and *MtTPR3a*. Different *cis*-elements including AW-box, AW-box-like, P1BS-box, CTTC/MYCS-box, GCC-box, and DRE-box motifs are represented by boxes in different colours[1–6]. The red line indicates the length of the promoters used in our *promoter:GUS* experiment.

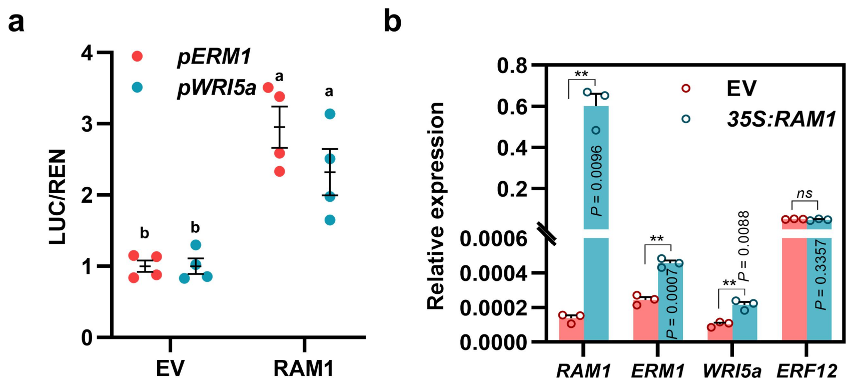

Supplementary Figure 36. RAM1 promotes the expression of *ERM1* and *WRI5a*. (**a**) *In vivo* transcriptional activation assay using a dual-luciferase system showing the activation of the 1-kb *ERM1* promoter and the 2-kb *WRI5a* promoter by RAM1 effector. (**b)** Relative expression levels of indicated genes in *M. truncatula* hairy roots (A17) transformed via *35S:RAM1* and EV. Relative expression was normalised to that of *MtEF-1*. Statistics: Individual data points and mean ± SE are shown. **a** $n = 4$ biological replicates. Different letters indicate significant differences (One-way ANOVA, Duncan's multiple range test, $P < 0.05$). Exact P values are provided in Source Data. **b** $n = 3$ technical replicates. Two-sided Student's t-test was used. $^{**}P < 0.01$; *ns*, not significant.

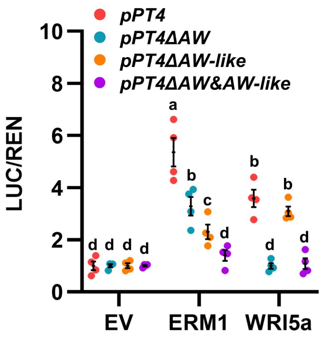

Supplementary Figure 37. *In vivo* transcriptional activation assay using a dual-luciferase system showing the activation of the 400-bp *PT4* promoters with various deletion of AW-box/AW-box-like elements by ERM1 or WRI5a, which acted as effectors. Statistics: Individual data points and mean ± SE are shown. $n = 4$ biological replicates. Different letters indicate significant differences (One-way ANOVA, Duncan's multiple range test, $P < 0.05$). Exact P values are provided in Source Data. This experiment was repeated twice with similar results.

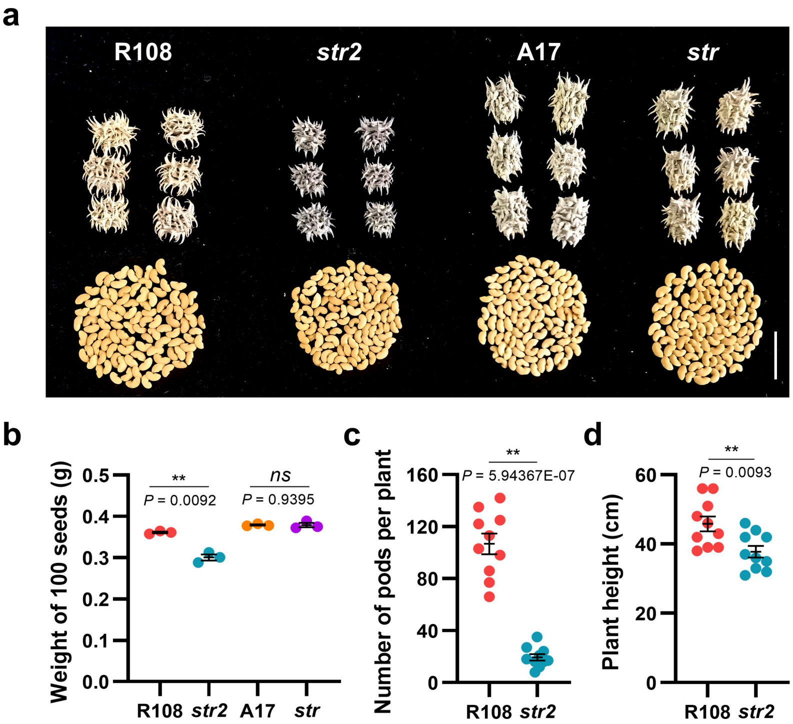

Supplementary Figure 38. The *str2* mutants exhibit pleiotropic developmental defects. (**a**) Morphology of pods [Number = 6] (up) and seeds [Number = 100] (down) for indicated genotypes. Scale bar, 1 cm. (**b-d**) The weight of 100 seeds (**b**), number of pods per plant (**c**), and plant height (**d**) for indicated genotypes. Statistics: Individual data points and mean ± SE are shown. **b** n = 3 biologically independent samples. **c**, **d** n = 10 biologically independent plants. Two-sided Student's *t*-test was used. **$P < 0.01$; *ns*, not significant. This experiment was repeated twice with similar results.

Supplementary Table 1. Expression profiles of *ERM1*, *ERF12*, and *TPL/TPR* genes in *Medicago* roots. Data were collected from the *M. truncatula* Gene Expression Atlas (*https://medicago.toulouse.inrae.fr/MtExpress*)[7].

Description Blast2n vs Mt genome 4.0 CDS	Noble Research Intitute legacy Gene Atlas V3 dataset	Non mycorrhizal cortical cell (rma)	Mycorrhizal cortical cell (rma)
***ERM1* (*Medtr6g012970*)**	**Mtr.21492.1.S1_at**	**12.7**	**96.8**
***ERF12* (*Medtr2g014300*)**	**Mtr.985.1.S1_at**	**471**	**1050**
MtTPL (*Medtr4g009840*)	Mtr.20141.1.S1_at	40.2	87.1
MtTPR1 (*Medtr2g104140*)	Mtr.40259.1.S1_s_at	92.2	69.8
MtTPR2 (*Medtr4g120900*)	Mtr.6940.1.S1_at	81.6	101
***MtTPR3a* (*Medtr7g112460*)**	**Mtr.23085.1.S1_s_at**	**95.9**	**262**
MtTPR3b (*Medtr1g083700*)	Mtr.49994.1.S1_s_at	85.2	152
MtTPR4 (*Medtr4g114980*)	Mtr.39072.1.S1_at	166	229
MtMtTPL-Like 1a (*Medtr2g435370*)	no hits	-	-
MtMtTPL-Like 1b (*Medtr2g065670*)	no hits	-	-
MtMtTPL-Like 2 (*Medtr1g012820*)	no hits	-	-

Supplementary Table 2. Primer sequences used in this study.

**Gene name**	**Primer sequences** (FP, forward primer; RP, reverse primer)	
**qRT-PCR Primers**		
WRI5a	FP	5'-AGAGGAGTAGCAAGGCACCATC-3'
	RP	5'- AGGTCCTGGCCTTAACCATCTT -3'
WRI5b	FP	5'-TGTACCAAAAATAGGTGATGATGCT-3'
	RP	5'-TCCATCTATGCCTGCTAACACC-3'
WRI5c	FP	5'-GCACCACCACAACGGTAGAT-3'
	RP	5'-TCCTCTTGGGTGCTGTAGGT-3'
MtPK	FP	5'-CACCAAGGGTCCTGAGGTTA-3'
	RP	5'-ACTTCGCATTTCACGGAATC-3'
MtKASII	FP	5'-GGGATAAAGACCGTGATGGAT-3'
	RP	5'-ACACACCGGCATCTACAAGAC-3'
MtKAR	FP	5'-TCCAAGGAGATTGAGGCACT-3'
	RP	5'-CCTCCTGCCACTGAGACTTC-3'
MtFatM	FP	5'-TTGAGCAAAGGCCAATAAGGT-3'
	RP	5'-CTATGTAGAAAATGGACATGTAGTGA-3'
RAM1	FP	5'-CTCATTCTCTTCGTATCCCCTTT-3'
	RP	5'-GTTTGGTGCTTGGTCTCTTATCAT-3'
RAM2	FP	5'-TTGGTGATGAAAAGCCTGAT-3'
	RP	5'-AAGATTATGGGTTTTGGAAGTTTG-3'
STR	FP	5'-TTCCAATGATGCAGTCCCA-3'
	RP	5'-TGGTTATGACTGCAAATGTGAG-3'
STR2	FP	5'-GCAAGTGGGAGTCTTAAAGGA-3'
	RP	5'-GCCCTAATCTGAAATCAGCAG-3'
PT4	FP	5'-GACACGAGGCGCTTTCATAGCAGC-3'
	RP	5'-GTCATCGCAGCTGGAACAGCACCG-3'
MtHA1	FP	5'-GACCATGTCTCTTGATGCTGT-3'
	RP	5'-CAGTTGCAATGACTATGGCCTC-3'
MtEF-1	FP	5'-CTTTGCTTGGTGCTGTTTAGATGG-3'
	RP	5'-ATTCCAAAGGCGGCTGCATA-3'
PP2A	FP	5'-GTCCTGGCGTGTGCGTTATATG-3'
	RP	5'-GGCACCAGATCCGTCCTAGTTG-3'
ERM1	FP	5'-CCTGATTTTCGGTTTCTCGACG-3'
	RP	5'-TGCTCTTCCATTTCCTCAGCA-3'
ERF12	FP	5'-GGCAGAACTATGGTGGCTGA-3'
	RP	5'-ATCGGAATCCCACGTCGAAC-3'
ERF12 Endo	FP	5'-TCCACCGTTGTGGCTTTGAA-3'
	RP	5'-TCCTAAAACGACGCCGTTAAAAA-3'
MtCBX1-like	FP	5'-TTGATTCTAACCCAACCTGTG-3'
	RP	5'-ATCTTGAGCATGGATGAAATT-3'
MtTPL	FP	5'-TTTTTCATGCGACAGCCAGC-3'
	RP	5'-TGCAGGAAGATAAGCAGGAGG-3'
MtTPR1	FP	5'-TACGTAATGTCGGCATCGGG-3'
	RP	5'-GTGGTGGCATGAACGTTGTC-3'
MtTPR2	FP	5'-CCTGGCCATTGTCGTGTAGT-3'
	RP	5'-CCCTTTAGCACCAAGAGCCA-3'
MtTPR3a	FP	5'-TTTTGCGATGGCAACACTGG-3'
	RP	5'-TGGCTTCCGCTTAAAGTCGT-3'
MtTPR3b	FP	5'-CATGCCTGACACCACAGACT-3'
	RP	5'-CAACACTGGCAGTTGCCTTC-3'
MtTPR4	FP	5'-CGCCTTCCCCTGTTACCAAT-3'
	RP	5'-GGGCCAAACCTATGGGTCC-3'

MtTPL-Like 1a	FP	5'-ACCTCGCTTGACGTTCAACA-3'
	RP	5'-TGAGCTATGGCAAATACAAACCT-3'
MtTPL-Like 1b	FP	5'-TTCGTGTGGGACAACTACGG-3'
	RP	5'-AACCGTTCACAGAAAGACCA-3'
MtTPL-Like 2	FP	5'-GCTCCAGCTTCTCCAGAACA-3'
	RP	5'-ACCACTCTTCCGATTCTCACG-3'
**cDNA primers**		
WRI5a	FP	5'-ATGGAGGAGGTTTCCAATGTGA-3'
	RP	5'-TCAGTTAGAAATGTTGGAAGGG -3'
WRI5b	FP	5'-ATGGCAATGTTGATAGAAAACGAA -3'
	RP	5'-TTATTGTCCAAAATTTAAGTA-3'
WRI5c	FP	5'-ATGGAAATGATGATGAAGGAA-3'
	RP	5'-CTAAGGTGTCCATTGGGGTTTCAG-3'
ERM1	FP	5'-ATGGCAAGGAAGAGAAAGGTTTCTGAAGCA-3'
	RP	5'-CTAATTGAATGAACCATTTTCAAGTAAGTT-3'
ERF12	FP	5'-ATGGCTTCTTCTTCAACTTCTTCAGCGAAT-3'
	RP	5'-TCAAAGCCACAACGGTGGAGGTTCATTCAA-3'
ERF12ΔEAR1	FP (P1)	5'-ATGGCTTCTTCTTCAACTTCTTCAGCGAAT-3'
	RP(P2)	5'-GCCATCGGAGATCAGAAGAACAAAGTCCTCCGGCGACC-3'
	FP (P3)	5'-GGTCGCCGGAGGACTTTGTTCTTCTGATCTCCGATGGC-3'
	RP(P4)	5'-TCAAAGCCACAACGGTGGAGGTTCATTCAA-3'
ERF12ΔEAR2	FP	5'-ATGGCTTCTTCTTCAACTTCTTCAGCGAAT-3'
	RP	5'-TCAAAGCCACAACGGAATCCCACGTCGAACAAGTCCCAAGTA-3'
ERF12ΔAP2	FP (P1)	5'-ATGGCTTCTTCTTCAACTTCTTCAGCGAAT-3'
	RP(P2)	5'-GAGATCCAAACAAAGTCCTCCTTCACGATTCGCTGA-3'
	FP (P3)	5'-TCAGCGAATCGTGAAGGAGGACTTTGTTTGGATCTC-3'
	RP(P4)	5'-TCAAAGCCACAACGGTGGAGGTTCATTCAA-3'
MtTPL	FP	5'-ATGTCATCTCTGAGTAGGGAATTGG-3'
	RP	5'-TCATCTTTGTGCTTGGTCTGAAGAA-3'
MtTPR1	FP	5'-ATGTTTGAAGCTTTTACCTTTGGAT-3'
	RP	5'-TCATCTTTGGACTTCATCAGAAGAA-3'
MtTPR2	FP	5'-ATGACATCTTTGAGTAGAGAATTGG-3'
	RP	5'-TCATCTTTGGAGCTGCTCAGAATTT-3'
MtTPR3a	FP	5'-ATGACTTCTTTGAGCAGAGAATTGG-3'
	RP	5'-TTATCTTTGTGTACCCTGATCGGGT-3'
MtTPR3b	FP	5'-ATGACTTCGTTGAGTAGAGAATTGG-3'
	RP	5'-TTATCGTTGGGCCTGATCCGCTGTG-3'
MtTPR4	FP	5'-ATGTCTTCGTTAAGCAGAGAACTCG-3'
	RP	5'-TCACCTTTGGGGTTGCTCTGAAACA-3'
RAM1	FP	5'-ATGATCAATTCACTTTGTGGAAGCT-3'
	RP	5'-TCAGCATCGCCATGCAGAAGCAGCG-3'
**RNAi primers**		
ERM1 RNAi-1	FP	5'-AAGAAGGAGCCCTTCACCATGGCAAGGAAGAGAAAGG-3'
	RP	5'-GTCGGCGCGCCCACCCTTATTTCGACGAAGAAAAGGAA-3'
ERM1 RNAi-2	FP	5'-AAGAAGGAGCCCTTCACCAGAGGAGCGTAGGGAGCAAC-3'
	RP	5'-GTCGGCGCGCCCACCCTTAGGTGAGTAGTAACTAGTTG-3'
ERF12 RNAi	FP	5'-AAGAAGGAGCCCTTCACCATGGCTTCTTCTTCAACTTC-3'
	RP	5'-GTCGGCGCGCCCACCCTTTAACTTCATTATGCCTCTGT-3'
WRI5a RNAi	FP	5'-AAGAAGGAGCCCTTCACCAGGCGGCGAAGAGAATCATC-3'
	RP	5'-GTCGGCGCGCCCACCCTTTAGAAAATCCACTACTCCTC-3'
ERM1-WRI5a-RNAi-1	FP (P1)	5'-AAGAAGGAGCCCTTCACCTAGGTGGCAATGAACAACAA-3'
	RP(P2)	5'-TTCCTTCTTCAACTGCTTCAGACAATGAAGCAAGATACTCTTCT-3'
	FP (P3)	5'-AGAAGAGTATCTTGCTTCATTGTCTGAAGCAGTTGAAGAAGGAA-3'
	RP(P4)	5'-GTCGGCGCGCCCACCCTTAAGTCTTTGAAGGAGGAGAT-3'

ERM1-WRI5a-RNAi-2	FP (P1)	5'-AAGAAGGAGCCCTTCACCTAGGTGGCAATGAACAACAA-3'
	RP(P2)	5'-TTGTTGCTCCCTACGCTCCTCTCAATGAAGCAAGATACTCTTCT-3'
	FP (P3)	5'-AGAAGAGTATCTTGCTTCATTGAGAGGAGCGTAGGGAGCAACAA-3'
	RP(P4)	5'-GTCGGCGCGCCCACCCTTAACACTTACATCATTATTGAC-3'
MtTPR3a RNAi	FP	5'-CTTTAAGAAGGAGCCCTTCACCTAAGAACGGTTGAAACTCCAGCATT-3'
	RP	5'-CTGGGTCGGCGCGCCCACCCTTTTCTGATCATTCCGAGACCACTTCC-3'
Complementation primers		
STR2	FP	5'-AAGAAGGAGCCCTTCACCAAATATCACAATAGACAAAGACATC-3'
	RP	5'-GTCGGCGCGCCCACCCTTCTAGGACCTTTGATTTTTTGATG-3'
ERM1	FP	5'-TTAAGAAGGAGCCCTTCACCGCCCTCTTATCACTTACGGAGCC-3'
	RP	5'-GGGTCGGCGCGCCCACCCTTCTAATTGAATGAACCATTTTCAAGT-3'
ERF12	FP	5'-TTAAGAAGGAGCCCTTCACCCCACTATTCATCTCCAATGACACATC-3'
	RP	5'-GTCGGCGCGCCCACCCTTTCAAAGCCACAACGGTGGAGGTTCA-3'
**Promoter primers**		
pSTR2 (1 kb)	FP	5'-TATTTTATTCTATTTAAGACATATT-3'
pSTR2 (250 bp)	FP	5'-AAACCAAGAATCCATTAGTTTCAAA-3'
pSTR2	RP	5'-TTTCTTGAGGTGCAAGTTAA-3'
pSTR2ΔAW-Box-like1 (250 bp)	FP	5'-ACACCAACCGGCCTTATCCTTATATACATCCT-3'
	RP	5'-AGGATGTATATAAGGATAAGGCCGGTTGGTGT-3'
pSTR2ΔAW-Box-like2 (250 bp)	FP	5'-AACAACATTGTAATAGTTACAATCTCCTGAAA-3'
	RP	5'-TTTCAGGAGATTGTAACTATTACAATGTTGTT-3'
pERM1 (1 kb)	FP	5'-TTAAGAAGGAGCCCTTCACCGCCCTCTTATCACTTACGGAGCC-3'
	RP	5'-GGGTCGGCGCGCCCACCCTTTATATGAACTCTTTTAAGCTCTT-3'
pERF12 (1 kb)	FP	5'-TTAAGAAGGAGCCCTTCACCTCGTAACTCTCTAACTTGTGCCT-3'
	RP	5'-GGGTCGGCGCGCCCACCCTTGAAAACAAAGAAAAACAACACAA-3'
pERF12ΔDRE (1 kb)	FP (P1)	5'-TTAAGAAGGAGCCCTTCACCTCGTAACTCTCTAACTTGTGCCT-3'
	RP (P2)	5'-ACCCACATTGGCTCCCGTAATTTGGCCGGCACCACTACAATACT-3'
	FP (P3)	5'-AGTATTGTAGTGGTGCCGGCCAAATTACGGGAGCCAATGTGGGT-3'
	RP (P4)	5'-GGGTCGGCGCGCCCACCCTTGAAAACAAAGAAAAACAACACAA-3'
pERF12ΔGCC (1 kb)	FP (P1)	5'-TTAAGAAGGAGCCCTTCACCTCGTAACTCTCTAACTTGTGCCT-3'
	RP (P2)	5'-AGTGGTGGGACCCTATTGGGATGGCTGAAAAGACGTATGGGGTG-3'
	FP (P3)	5'-CACCCCATACGTCTTTTCAGCCATCCCAATAGGGTCCCACCACT-3'
	RP (P4)	5'-GGGTCGGCGCGCCCACCCTTGAAAACAAAGAAAAACAACACAA-3'
*pERF12Δ*AW (1 kb)	FP (P1)	5'-TTAAGAAGGAGCCCTTCACCTCGTAACTCTCTAACTTGTGCCT-3'
	RP (P2)	5'-AGTTTATTTGGATGAAATAGTTTAACATGCGGTGTTTTTTATTT-3'
	FP (P3)	5'-AAATAAAAAACACCGCATGTTAAACTATTTCATCCAAATAAACT-3'
	RP (P4)	5'-GGGTCGGCGCGCCCACCCTTGAAAACAAAGAAAAACAACACAA-3'
pWRI5a (2 kb)	FP	5'-TTATTGATTATACTTTTTTAGACCA-3'
	RP	5'-TGATCAATACTCTTCACTTTCTTCG-3'
pPT4 (400 bp)	FP	5'-TTAAGAAGGAGCCCTTCACCTCACTTTCTTAATTCCTGTGAT-3'
	RP	5'-GGTCGGCGCGCCCACCCTTTTTGGTAAAAAAAAAAAGGAAGACTCT-3'
pPT4ΔAW (400 bp)	FP (P1)	5'-TTAAGAAGGAGCCCTTCACCTCACTTTCTTAATTCCTGTGAT-3'
	RP (P2)	5'-TGTAACAATGTGACGTTTAGTCTTAGCTTAGGAGATGAG-3'
	FP (P3)	5'-CTCATCTCCTAAGCTAAGACTAAACGTCACATTGTTACA-3'
	RP (P4)	5'-GGTCGGCGCGCCCACCCTTTTTGGTAAAAAAAAAAAGGAAGACTCT-3'
pPT4ΔAW-Box-like (400 bp)	FP (P1)	5'-TTAAGAAGGAGCCCTTCACCTCACTTTCTTAATTCCTGTGAT-3'
	RP (P2)	5'-AGCTAAATGTTATGGATATTCATGTTGATCAATTAATTA-3'
	FP (P3)	5'-TAATTAATTGATCAACATGAATATCCATAACATTTAGCT-3'
	RP (P4)	5'-GGTCGGCGCGCCCACCCTTTTTGGTAAAAAAAAAAAGGAAGACTCT-3'
pMtTPL (1 kb)	FP	5'-GCTTATGGGCCTCACCATATTGCATCAGC-3'
	RP	5'-CTTCAACACACTAAAATCTGCAATAC-3'
pMtTPR1 (2.1 kb)	FP	5'-ACGACTCTTTTTAATGTGCTGACAAT-3'

	RP	5'-GGAGAAAGAAAACAACAACCACTTCA-3'
pMtTPR3a (1.6 kb)	FP	5'-ATGGTGATTCATGGTGGAACAAATGA-3'
	RP	5'-AGCGGATCCTCAAATCTGAACACCGAT-3'
***Tnt 1* mutant primers**		
Tnt1-F2		5'-TCTTGTTAATTACCGTATCTCGGTGCTACA-3'
*NF11835*_F1	FP	5'-TCACAATCCACCAACCTTCA-3'
*NF11835*_R1	RP	5'-AGACATGGATCCTAATGCGC-3'
*NF16266*_F1	FP	5'-CTAAGGTTCACTCACTCTCCCTTC-3'
*NF16266*_R1	RP	5'-TCTTCCATTTCCTCAGCAATCTCA-3'
*NF19239*_F2	FP	5'-CAAACACCGTACAACAACAGTAGG-3'
*NF19239*_R1	RP	5'-TCTAGGGTTTTTCAAAGCCACAAC-3'
**Y1H/Y2H**		
WRI5a	FP	5'-GAATTCATGGAGGAGGTTTCCAATGT-3'
	RP	5'-GGATCCTCAGTTAGAAATGTTGGAAGGG-3'
WRI5b	FP	5'-CGCGGATCCATGGCAATGTTGATAGAAAACGAA-3'
	RP	5'-CCGGAGCTCTTATTGTCCAAAATTTAAGTATTG-3'
WRI5c	FP	5'-CGCGGATCCATGGAAATGATGATGAAGGAA-3'
	RP	5'-CCGGAGCTCCTAAGGTGTCCATTGGGG-3'
ERM1	FP	5'-AAGAAGGAGCCCTTCACCATGGCAAGGAAGAGAAAGGTTTCTG-3'
	RP	5'-GTCGGCGCGCCCACCCTTCTAATTGAATGAACCATTTTCAAGT-3'
ERF12	FP	5'-AAGAAGGAGCCCTTCACCATGGCTTCTTCTTCAACTTCTTCAG-3'
	RP	5'-GTCGGCGCGCCCACCCTTTCAAAGCCACAACGGTGGAGGTTCA-3'
MtTPL	FP	5'-CTTTAAGAAGGAGCCCTTCACCATGTCATCTCTGAGTAGGGAATTGG-3'
	RP	5'-CTGGGTCGGCGCGCCCACCCTTTCATCTTTGTGCTTGGTCTGAAGAA-3'
MtTPR1	FP	5'-CTTTAAGAAGGAGCCCTTCACCATGTTTGAAGCTTTTACCTTTGGAT-3'
	RP	5'-CTGGGTCGGCGCGCCCACCCTTTCATCTTTGGACTTCATCAGAAGAA-3'
MtTPR2	FP	5'-CTTTAAGAAGGAGCCCTTCACCATGACATCTTTGAGTAGAGAATTGG-3'
	RP	5'-CTGGGTCGGCGCGCCCACCCTTTCATCTTTGGAGCTGCTCAGAATTT-3'
MtTPR3a	FP	5'-CTTTAAGAAGGAGCCCTTCACCATGACTTCTTTGAGCAGAGAATTGG-3'
	RP	5'-CTGGGTCGGCGCGCCCACCCTTTTATCTTTGTGTACCCTGATCGGGT-3'
MtTPR3b	FP	5'-CTTTAAGAAGGAGCCCTTCACCATGACTTCGTTGAGTAGAGAATTGG-3'
	RP	5'-CTGGGTCGGCGCGCCCACCCTTTTATCGTTGGGCCTGATCCGCTGTG-3'
MtTPR4	FP	5'-CTTTAAGAAGGAGCCCTTCACCATGTCTTCGTTAAGCAGAGAACTCG-3'
	RP	5'-CTGGGTCGGCGCGCCCACCCTTTCACCTTTGGGGTTGCTCTGAAACA-3'
250 bp *pSTR*	FP	5'-CCGGAATTCTGAGTGAAACAAAACTACGTC-3'
	RP	5'-GCCGAGCTCTGCGTAGTAGAGCAATCAC-3'
250 bp *pSTR2*	FP	5'-GAATTCCCGGGGAGCTCAAACCAAGAATCCATTAGTTTCAAA-3'
	RP	5'-CGCGGATCGATTCGCGATTTCTTGAGGTGCAAGTTAAGTT-3'
1 kb *pERF12*	FP	5'-GAATTCCCGGGGAGCTCTCGTAACTCTCTAACTTGTGCCT-3'
	RP	5'-CGCGGATCGATTCGCGAGAAAACAAAGAAAAACAACACAA-3'
**BiFC primers**		
ERM1-pXY104	FP	5'-GGATCCATGGCAAGGAAGAGAAAGGTTTCTGAAGCA-3'
ERM1-pXY104	RP	5'-GTCGACATTGAATGAACCATTTTCAAGTAAGTT-3'
ERM1-pXY106	FP	5'-GGATCCATGGCAAGGAAGAGAAAGGTTTCTGAAGCA-3'
ERM1-pXY106	RP	5'-GTCGACCTAATTGAATGAACCATTTTCAAGTAAGTT-3'
WRI5a-pXY104	FP	5'-GGATCCATGGAGGAGGTTTCCAATGT-3'
WRI5a-pXY104	RP	5'-GTCGACGTTAGAAATGTTGGAAGGG-3'
WRI5a-pXY106	FP	5'-GGATCCATGGAGGAGGTTTCCAATGT-3'
WRI5a-pXY106	RP	5'-GTCGACTTAGTTAGAAATGTTGGAAGGG-3'
ERF12-pXY104	FP	5'-GGATCCATGGCTTCTTCTTCAACTTCTTCAGCGAAT-3'
ERF12-pXY104	RP	5'-GTCGACAAGCCACAACGGTGGAGGTTCATTCAA-3'
ERF12-pXY106	FP	5'-GGATCCATGGCTTCTTCTTCAACTTCTTCAGCGAAT-3'
ERF12-pXY106	RP	5'-GTCGACTTAAAGCCACAACGGTGGAGGTTCATTCAA-3'
STR-pXY106	FP	5'-CAACATCGAGGACGCCGGCGGATCCATGGCAAGGCTCGAGAGGGAT-3'

STR-pXY106	RP	5'-ACGAACGAAAGCTCTGCAGGTCGACTCATTTTCTTTCATTTTTGGAG-3'
STR2-pXY104	FP	5'-TACAATTACAGGTACCCGGGGATCCATGAAAACACAAGGTCTTGAA-3'
STR2-pXY104	RP	5'-GCTGCACGCTGCCACCGCCGTCGACGGACCTTTGATTTTTTGATGCA-3'
STR2-pXY106	FP	5'-CAACATCGAGGACGCCGGCGGATCCATGAAAACACAAGGTCTTGAA-3'
STR2-pXY106	RP	5'-ACGAACGAAAGCTCTGCAGGTCGACGGACCTTTGATTTTTTGATGCA-3'
ERF12ΔEAR1-pXY104	FP	5'-GGATCCATGGCTTCTTCTTCAACTTCTT-3'
ERF12ΔEAR1-pXY104	RP	5'-GTCGACAAGCCACAACGGTGGAGGTTCA-3'
ERF12ΔEAR1-pXY106	FP	5'-GGATCCATGGCTTCTTCTTCAACTTCTT-3'
ERF12ΔEAR1-pXY106	RP	5'-GTCGACTCAAAGCCACAACGGTGGAGGT-3'
ERF12ΔEAR2-pXY104	FP	5'-GGATCCATGGCTTCTTCTTCAACTTCTT-3'
ERF12ΔEAR2-pXY104	RP	5'-GTCGACAAGCCACAACGGAATCCCACGTC-3'
ERF12ΔEAR2-pXY106	FP	5'-GGATCCATGGCTTCTTCTTCAACTTCTT-3'
ERF12ΔEAR2-pXY106	RP	5'-GTCGACTCAAAGCCACAACGGAATCCCAC-3'
ERF12ΔAP2-pXY104	FP	5'-GGATCCATGGCTTCTTCTTCAACTTCTT-3'
ERF12ΔAP2-pXY104	RP	5'-GTCGACAAGCCACAACGGTGGAGGTTCA-3'
ERF12ΔAP2-pXY106	FP	5'-GGATCCATGGCTTCTTCTTCAACTTCTT-3'
ERF12ΔAP2-pXY106	RP	5'-GTCGACTCAAAGCCACAACGGTGGAGGT-3'
**ChIP-qPCR primers**		
IP-STR-1-F	FP	5'-TCTGGTGCTGAGTGAAACA-3'
IP-STR-1-R	RP	5'-AGAGGCAGGTGATTAAATATGG-3'
IP-STR-2-F	FP	5'-GTTGAATGCACGGTTTTTGA-3'
IP-STR-2-R	RP	5'-CGTGCCTTCAAAGATGTGTCT-3'
IP-STR-3-F	FP	5'-CTCGATGAGCCGGATCTTGG-3'
IP-STR-3-R	RP	5'-CGAAGAGTTCTGGAGTGCGA-3'
IP-STR2-1-F	FP	5'-GTATTCTTTCAATGAAAACAACATTGT-3'
IP-STR2-1-R	RP	5'-GTGTGCTGAGGATGTATATA-3'
IP-STR2-2-F	FP	5'-GGAGATGAAGGCACAAGAGGA-3'
IP-STR2-2-R	RP	5'-GTCCTGAAGTTGGTTCATCTAGGA-3'
IP-ERF12-1-F	FP	5'-AGTATTGTAGTGGTGCCGGC-3'
IP-ERF12-1-R	RP	5'-AGGGTTTGCGAAGTGAAGTGA-3'
IP-ERF12-2-F	FP	5'-ACAGTAGGGCATACGTCACG-3'
IP-ERF12-2-R	RP	5'-TGAAAGAAAGTGGTGGGACCC-3'
IP-ERF12-3-F	FP	5'-ACACCGCATGTTACATCCTTCA-3'
IP-ERF12-3-R	RP	5'-TGCTAATGTTAAATTTCGTTGGTGA-3'
IP-ERF12-4-F	FP	5'-CCATCTGTCCCTTCACGTGG-3'
IP-ERF12-4-R	RP	5'-CTCTGTCCAACCACCCCG-3'
IP-EF-1-F	FP	5'-CAGCAAGGACACTTTTGG-3'
IP-EF-1-R	RP	5'-CTCAGCTGAATCGAAATCTAG-3'

Supplementary Table 3. The generation of plasmids used in this study. Primer sequences used for plasmid construction are listed in Supplementary Table 2.

Purpose	Construction	Description
Co-overexpression	*STR*/*STR2* co-overexpression	A binary vector for *STR* and *STR2* co-overexpression analysis in *A. thaliana wbc11* used in this study has been described previously[8]. Briefly, the cDNA sequences of *STR* and *STR2* were amplified by PCR and cloned into pENTR/SD/D-Topo and pDNOR-p4p3 (Invitrogen), respectively. These two fragments were then transferred from the entry vectors into pK7m34GW2-8m21GW3D (containing 835 bp *35S* promoter, ROLD promoter and GFP fluorescence marker) by Gateway LR reactions.
Overexpression analysis	*35S:ERM1*, *35S:ERF12*, *35S:WRI5a*, *35S:RAM1*, *35S:ERF12ΔEAR1*, *35S:ERF12ΔEAR2*, *35S:ERF12ΔEAR1ΔEAR2*, *35S:ERF12ΔAP2*	The cDNA sequences of *ERM1* (1,236 bp), *ERF12* (519 bp), *WRI5a* (1,203 bp), *RAM1* (2,025 bp), *ERF12△EAR1* (501 bp), *ERF12ΔEAR2* (501 bp), *ERF12ΔEAR1ΔEAR2* (477 bp), and *ERF12ΔAP2* (327 bp) were amplified by PCR and cloned into pENTR/SD/D-Topo (Invitrogen). Each fragment was then transferred from the entry vectors into pK7WG2R by Gateway LR reactions (Invitrogen).
	pPT4:ERM1, *pPT4:ERF12*, *pPT4:MtTPR3a*	The *35S* promoter of pK7WG2R was removed by *Hind*III/*Spe*I digestion and replaced by an 863 bp *PT4* promoter, resulting in *pPT4:*pK7WG2Rδ35S. The *ERM1*, *ERF12*, and *MtTPR3a* cDNA fragment (cloned into pENTR/SD/D-Topo) was then transferred from the entry vectors into *p PT4:*pK7WG2Rδ35S by Gateway LR reactions.
RNAi analysis	*ERM1*-RNAi-1, *ERM1*-RNAi-2, *ERF12*-RNAi, *WRI5a*-RNAi, *ERM1-WRI5a*-RNAi-1, *ERM1-WRI5a*-RNAi-2, *MtTPR3a*-RNAi-1, *MtTPR3a*-RNAi-2	The RNAi target regions of two 400 bp *ERM1* cDNA sequences (*ERM1*-RNAi-1 and *ERM1*-RNAi-2), a 400 bp *ERF12* cDNA sequence (*ERF12*-RNAi), a 400 bp *WRI5a* cDNA sequence (*WRI5a*-RNAi), and two 400 bp *MtTPR3a* cDNA sequences (*MtTPR3a*-RNAi-1 and *MtTPR3a*-RNAi-2) were amplified by PCR and cloned into pENTR/SD/D-Topo, respectively. For *ERM1* and *WRI5a* fusion RNAi analysis, the RNAi target sequences of both two genes were amplified by PCR, overlapped and cloned into pENTR/SD/D-Topo. Each fragment was then transferred into pK7GWIWGIIR by Gateway LR reactions.
Promoter-*GUS* analysis	1 kb p*STR2:GUS*, 250 bp p*STR2:GUS*, 250 bp p*STR2ΔAW-Box-like1:GUS*, 250 bp p*STR2ΔAW-Box-like2:GUS*, 1 kb p*ERM1:GUS*, 1 kb p*ERF12:GUS*, 1 kb p*MtTPL:GUS*, 2.1 kb p*MtTPR1:GUS*, 1.6 kb p*MtTPR3a:GUS*	The 5' flanking genomic sequences of *STR2* (different lengths including -1/-1000 bp, -1/-250 bp upstream of ATG start codon and forms including -1/-250 bp with AW-box-like1, AW-box-like2, AW-box-like1&2 deletion upstream of ATG start codon), *ERM1* (1 kb), *ERF12* (1 kb), *MtTPL* (1 kb), *MtTPR1* (2.1 kb), and *MtTPR3a* (1.6 kb) were amplified by PCR and cloned into pENTR/SD/D-Topo. The fragments were then transferred from the entry vectors into pBGWFS7 by Gateway LR reactions.
Complementation analysis	p*STR2:STR2*, p*ERM1:ERM1*, p*ERF12:ERF12*	The 35S promoter of pK7WG2R was removed by *Hind*III/*Spe*I digestion and replaced by a 2 kb of the *STR2* promoter, resulting in *pSTR2:*pK7WG2Rδ35S. A pENTR/SD/D-Topo clone containing a cDNA fragment of *STR2* was then transferred from the entry vectors into *pSTR2:*pK7WG2Rδ35S by Gateway LR reactions, resulting in 2 kb *pSTR2:STR2*. The 1 kb *pERM1:ERM1* and 2 kb *pERF12:ERF12* vectors were generated in the same way.
Y1H/Y2H	p*STR2*-pHIS2, p*ERF12*-pHIS2, WRI5a/WRI5b/WRI5c/ERM1/ERF12/MtTPL/MtTPR1/MtTPR2/MtTPR3a/MtTPR3b/MtTPR4-pGADT7/pGBKT7	The 250 bp *STR2* promoter and 1,000 bp *ERF12* promoter fragments were inserted into the pHIS2 vector. The target coding sequences of WRI5a, WRI5b, WRI5c, ERM1, ERF12, MtTPL, MtTPR1, MtTPR2, MtTPR3a, MtTPR3b, and MtTPR4 were cloned into the pGADT7-GW AD and pGBKT7-GW BD vectors, respectively.
BiFC analysis	STR/STR2/ERM1/ERF12/ERF12ΔEAR1/ERF12ΔEAR2/ERF12ΔAP2/WRI5a-pXY106, STR2/ERM1/ERF12/ERF12ΔEAR1/ERF12ΔEAR2/ERF12ΔAP2/WRI5a-pXY104	The coding sequence of STR, STR2, ERM1, ERF12, domain-deleted ERF12 (ERF12ΔEAR1/ERF12ΔEAR2/ERF12ΔAP2), and WRI5a were amplified by PCR and cloned into the pXY106 and pXY104 vectors, respectively.
Transactivation assay and subcellular localisation analysis	ERM1/ERF12/WRI5a/RAM1-pGWB441, p*STR*/p*STRΔAW1*/p*STRΔAW2*/p*STRΔAW1&2*/p*STR2*/p*STR2ΔAW-like1*/p*STR2ΔAW-like2*/p*STR2ΔAW-like1&2*/p*ERF12*/p*ERF12ΔDRE*/p*ERF12ΔGCC*/p*ERF12ΔAW*/p*PT4*/p*PT4ΔAW*/p*PT4ΔAW-like*/p*PT4ΔAW&AW-like*-pGreenII-0800-LUC	The *ERM1*, *ERF12*, *WRI5a*, and *RAM1* cDNA sequences were amplified by PCR and cloned into the 35S-C-EYFP binary vector pGWB441.<br>The 250 bp *STR* promoter sequences (with various deletion of ΔAW1/ΔAW2/ΔAW1&2 elements), 250 bp *STR2* promoter sequences (with various deletion of ΔAW-like1/ΔAW-like2/ΔAW-like1&2 elements), 1 kb *ERF12* promoter sequences (with various deletion of ΔDRE/ΔGCC/ΔAW elements), and 400 bp *PT4* promoter sequences (with various deletion of ΔAW/ΔAW-like/ΔAW&AW-like elements) were amplified by PCR and cloned into the pGreenII-0800-LUC vector, respectively.

ChIP assays	ERM1/ERF12/ WRI5a-3×FLAG-pK7WG2R	The 3×FLAG sequence was amplified by PCR and cloned into pENTR/SD/D-Topo (Invitrogen). The cDNA sequences (without terminator) of *ERM1*, *ERF12*, and *WRI5a* were amplified by PCR and inserted into the above-mentioned modified pENTR/SD/D-Topo entry vectors. Each fragment was then transferred from the entry vectors into pK7WG2R by Gateway LR reactions.

Supplementary References

1. Jiang, Y. et al. *Medicago* AP2-domain transcription factor WRI5a is a master regulator of lipid biosynthesis and transfer during mycorrhizal symbiosis. *Mol. Plant* **11**, 1344–1359 (2018).
2. Xue, L. et al. AP2 transcription factor CBX1 with a specific function in symbiotic exchange of nutrients in mycorrhizal Lotus japonicus. *Proc. Natl Acad. Sci. USA* **115**, E9239–E9246 (2018).
3. Fujimoto, S. Y., Ohta, M., Usui, A., Shinshi, H. & Ohme-Takagi, M. *Arabidopsis* ethylene-responsive element binding factors act as transcriptional activators or repressors of GCC box-mediated gene expression. *Plant Cell* **12**, 393–404 (2000).
4. Song, C. P. et al. Role of an *Arabidopsis* AP2/EREBP-type transcriptional repressor in abscisic acid and drought stress responses. *Plant Cell* **17**, 2384–2396 (2005).
5. Li, X. et al. ETR1/RDO3 regulates seed dormancy by relieving the inhibitory effect of the ERF12-TPL complex on *DELAY OF GERMINATION1* expression. *Plant Cell* **31**, 832–847 (2019).
6. Shi, J. et al. A phosphate starvation response-centered network regulates mycorrhizal symbiosis. *Cell* **184**, 5527–5540.e5518 (2021).
7. Carrere S, Verdier J, Gamas P, MtExpress, a Comprehensive and Curated RNAseq-based Gene Expression Atlas for the Model Legume *Medicago truncatula*. *Plant Cell Physiol*. **62**, 1494-1500 (2021).
8. Jiang, Y. et al. Plants transfer lipids to sustain colonization by mutualistic mycorrhizal and parasitic fungi. *Science* **356**, 1172–1175 (2017).
