# Supplementary material for: Control of arbuscule development by a transcriptional negative feedback loop in Medicago
Source: Nat Commun. 2023 Sep 16;14:5743. doi: 10.1038/s41467-023-41493-2 (PMC10505183; doi:10.1038/s41467-023-41493-2)
Supplement: Supplementary file 2 — Reporting Summary [file 41467_2023_41493_MOESM2_ESM.pdf]

nature portfolio

Corresponding author(s): Yina Jiang, Ertao Wang

Last updated by author(s): Aug 29, 2023

Reporting Summary

Nature Portfolio wishes to improve the reproducibility of the work that we publish. This form provides structure for consistency and transparency in reporting. For further information on Nature Portfolio policies, see our Editorial Policies and the Editorial Policy Checklist.

Statistics

For all statistical analyses, confirm that the following items are present in the figure legend, table legend, main text, or Methods section.

n/a	Confirmed	
☐	☒	The exact sample size (n) for each experimental group/condition, given as a discrete number and unit of measurement
☐	☒	A statement on whether measurements were taken from distinct samples or whether the same sample was measured repeatedly
☐	☒	The statistical test(s) used AND whether they are one- or two-sided<br>*Only common tests should be described solely by name; describe more complex techniques in the Methods section.*
☒	☐	A description of all covariates tested
☐	☒	A description of any assumptions or corrections, such as tests of normality and adjustment for multiple comparisons
☐	☒	A full description of the statistical parameters including central tendency (e.g. means) or other basic estimates (e.g. regression coefficient) AND variation (e.g. standard deviation) or associated estimates of uncertainty (e.g. confidence intervals)
☐	☒	For null hypothesis testing, the test statistic (e.g. F, t, r) with confidence intervals, effect sizes, degrees of freedom and P value noted<br>*Give P values as exact values whenever suitable.*
☒	☐	For Bayesian analysis, information on the choice of priors and Markov chain Monte Carlo settings
☒	☐	For hierarchical and complex designs, identification of the appropriate level for tests and full reporting of outcomes
☒	☐	Estimates of effect sizes (e.g. Cohen's d, Pearson's r), indicating how they were calculated

Our web collection on statistics for biologists contains articles on many of the points above.

Software and code

Policy information about availability of computer code

Data collection	Olympus MVX10 fluorescence microscope (RCL quantification); Zeiss Axio Imager A2 light microscope (Germany) & Zeiss ZEN 2.5 lite (Promoter:GUS imaging); Leica SP8 confocal microscope (AM imaging); Bio-Rad CFX Maestro 1.1 (RT-qPCR, ChIP-qPCR); MEGA 11 (Phylogenetic tree); Synergy 2 multimode microplate reader (Bio-Tek) (Transactivation assay); Tanon 4600 Automatic chemiluminescence image analysis system (Shanghai, China) (Western Blotting)
Data analysis	GraphPad Prism v8.0 (AM colonisation, qPCR, transactivation assay, BiFC intensity, cutin/FA measurement); Leica Application Suite X (3.3.0) software (BiFC signal quantification); MassHunter Quantitative and Qualitative Analysis version B07 & National Institute of Standards and Technology library (NIST 14) (GC-MS assay); MEGA 4.0.2 software (Phylogenetic analysis); ImageJ software (arbuscule size quantification); DNAMAN 7.0 software (Sequence alignment)

For manuscripts utilizing custom algorithms or software that are central to the research but not yet described in published literature, software must be made available to editors and reviewers. We strongly encourage code deposition in a community repository (e.g. GitHub). See the Nature Portfolio guidelines for submitting code & software for further information.

Data

Policy information about availability of data

All manuscripts must include a data availability statement. This statement should provide the following information, where applicable:

- Accession codes, unique identifiers, or web links for publicly available datasets
- A description of any restrictions on data availability
- For clinical datasets or third party data, please ensure that the statement adheres to our policy

The data that support the findings of this study are available in the main text or supplementary information. The M . truncatula Tnt1 transposon insertion lines used in this study were obtained from the Medicago truncatula Mutant Database (https://medicago-mutant.dasnr.okstate.edu/mutant/index.php). Source data are provided with this paper.

Human research participants

Policy information about studies involving human research participants and Sex and Gender in Research.

Reporting on sex and gender	*Use the terms sex (biological attribute) and gender (shaped by social and cultural circumstances) carefully in order to avoid confusing both terms. Indicate if findings apply to only one sex or gender; describe whether sex and gender were considered in study design whether sex and/or gender was determined based on self-reporting or assigned and methods used. Provide in the source data disaggregated sex and gender data where this information has been collected, and consent has been obtained for sharing of individual-level data; provide overall numbers in this Reporting Summary. Please state if this information has not been collected. Report sex- and gender-based analyses where performed, justify reasons for lack of sex- and gender-based analysis.*
Population characteristics	*Describe the covariate-relevant population characteristics of the human research participants (e.g. age, genotypic information, past and current diagnosis and treatment categories). If you filled out the behavioural & social sciences study design questions and have nothing to add here, write "See above."*
Recruitment	*Describe how participants were recruited. Outline any potential self-selection bias or other biases that may be present and how these are likely to impact results.*
Ethics oversight	*Identify the organization(s) that approved the study protocol.*

Note that full information on the approval of the study protocol must also be provided in the manuscript.

Field-specific reporting

Please select the one below that is the best fit for your research. If you are not sure, read the appropriate sections before making your selection.

- [x] Life sciences
- [ ] Behavioural & social sciences
- [ ] Ecological, evolutionary & environmental sciences

For a reference copy of the document with all sections, see nature.com/documents/nr-reporting-summary-flat.pdf

Life sciences study design

All studies must disclose on these points even when the disclosure is negative.

Sample size	No statistical analyses were performed to predetermine sample size in any of the experiment. Sample sizes were chosen according to the standard for these types of analyses. In RCL quantification assays, 8-16 biological replicates (represented by one plant each) were used. In transactivation assays, 3-4 biological replicates were collected. These represent 3-4 independent Agrobacterium infiltrations, one per leaf into leaves of 4 individual plants. In BiFC intensity assays, 30 cells for each BiFC combination were used. In qPCR assays, 3 technical or biological replicates were used as indicated. In pro:GUS assays, 5-10 biological replicates (represented by one plant each) were used. The sample size of any other experiments has been indicated in the figure legends.
Data exclusions	No data presented or mentioned in this work has been excluded.
Replication	All attempts at replication were successful.
Randomization	Samples were randomly allocated to each group and treated.
Blinding	All experiments were blinded during data acquisition and analyses.

Reporting for specific materials, systems and methods

We require information from authors about some types of materials, experimental systems and methods used in many studies. Here, indicate whether each material, system or method listed is relevant to your study. If you are not sure if a list item applies to your research, read the appropriate section before selecting a response.

Materials & experimental systems

n/a	Involved in the study
☐	☒ Antibodies
☒	☐ Eukaryotic cell lines
☒	☐ Palaeontology and archaeology
☒	☐ Animals and other organisms
☒	☐ Clinical data
☒	☐ Dual use research of concern

Methods

n/a	Involved in the study
☒	☐ ChIP-seq
☒	☐ Flow cytometry
☒	☐ MRI-based neuroimaging

Antibodies

Antibodies used	Anti-FLAG M2 Magnetic Beads (8823, SIGMA); IgG (ab171870, Abcam); Antibody dilution used for each antibody was 1:200.<br>Anti-GAL4-BD (Abbkine, Cat#ABP57232, 1:2,000).<br>Goat anti-rabbit IgG secondary antibody (Thermo Fisher, Cat#31460, 1:10,000).
Validation	Both the Anti-FLAG and IgG antibody as described above were previously used (Reference 16; Jiang et al., 2018) for ChIP assays involving the same transgenic line UBQ:WRI5a-FLAG as used in this study.<br>Anti-GAL4-BD (https://www.abbkine.cn/product/abp57232/).<br>Goat anti-Rabbit IgG Secondary Antibody (https://www.thermofisher.cn/cn/zh/antibody/product/Goat-anti-Rabbit-IgG-H-L-Secondary-Antibody-Polyclonal/31460).
